# Craniofacial dysmorphology in Down syndrome is caused by increased dosage of Dyrk1a and at least three other genes

**DOI:** 10.1242/dev.201077

**Published:** 2023-04-26

**Authors:** Yushi Redhead, Dorota Gibbins, Eva Lana-Elola, Sheona Watson-Scales, Lisa Dobson, Matthias Krause, Karen J. Liu, Elizabeth M. C. Fisher, Jeremy B. A. Green, Victor L. J. Tybulewicz

**Affiliations:** ^1^ Centre for Craniofacial Biology and Regenerative Biology, King's College London, London SE1 9RT, UK; ^2^ The Francis Crick Institute, London NW1 1AT, UK; ^3^ Randall Centre for Cell and Molecular Biophysics, King's College London, London SE1 1UL, UK; ^4^ Institute of Neurology, University College London, London WC1N 3BG, UK

**Keywords:** Down syndrome, Craniofacial development, *Dyrk1a*, Neural crest, Morphometrics, Synchondroses

## Abstract

Down syndrome (DS), trisomy of human chromosome 21 (Hsa21), occurs in 1 in 800 live births and is the most common human aneuploidy. DS results in multiple phenotypes, including craniofacial dysmorphology, which is characterised by midfacial hypoplasia, brachycephaly and micrognathia. The genetic and developmental causes of this are poorly understood. Using morphometric analysis of the Dp1Tyb mouse model of DS and an associated mouse genetic mapping panel, we demonstrate that four Hsa21-orthologous regions of mouse chromosome 16 contain dosage-sensitive genes that cause the DS craniofacial phenotype, and identify one of these causative genes as *Dyrk1a*. We show that the earliest and most severe defects in Dp1Tyb skulls are in bones of neural crest (NC) origin, and that mineralisation of the Dp1Tyb skull base synchondroses is aberrant. Furthermore, we show that increased dosage of *Dyrk1a* results in decreased NC cell proliferation and a decrease in size and cellularity of the NC-derived frontal bone primordia. Thus, DS craniofacial dysmorphology is caused by an increased dosage of *Dyrk1a* and at least three other genes.

## Teaser

**Summary:** Craniofacial dysmorphology in mouse models of Down syndrome is caused by increased dosage of at least four genes, including *Dyrk1a*, resulting in reduced proliferation of neural crest-derived cranial bone progenitors.

## INTRODUCTION

Down syndrome (DS), trisomy 21, is caused by an additional third copy of human chromosome 21 (Hsa21). DS is the most common human aneuploidy, occurring in 1 in 800 live births, and results in numerous phenotypes, including learning and memory deficits, heart defects and early onset Alzheimer's disease (Antonarakis, 2017; Bergström et al., 2016; Lott and Dierssen, 2010; Wiseman et al., 2015). Individuals with DS also have a characteristic craniofacial dysmorphology with a near 100% penetrance, characterised by reduction in the dimensions of the midface, with flattened nose bridge (midfacial hypoplasia), shortening of the skull along the anteroposterior axis (brachycephaly), reduction in the dimensions of the lower jaw (micrognathia), altered shape of the skull orbit and an absence or reduction of permanent teeth (hypodontia) (Antonarakis, 2017; Vicente et al., 2020).

DS is a gene dosage disorder with a third copy of one or more of the ∼230 genes on Hsa21 causing the multiple phenotypes. However, the identity of the causative dosage-sensitive genes is largely unknown and thus the pathological mechanisms underlying DS phenotypes are unclear. Knowledge of such genes and mechanisms is essential for targeted therapies, as there are no treatments for most aspect of DS. In particular, the genes and mechanisms driving craniofacial dysmorphology in DS are poorly understood.

The search for dosage-sensitive genes causing DS phenotypes has used both human and mouse genetics. Analysis of rare partial trisomies of Hsa21 has been used to map individual phenotypes to specific regions of the chromosome, initially to the so-called Down syndrome critical region (DSCR) (Delabar et al., 1993; Korenberg et al., 1994; McCormick et al., 1989), but more recently to multiple regions of the chromosome (Korbel et al., 2009; Lyle et al., 2009). As these partial trisomies are rare, there is insufficient genetic resolution to identify causative genes using this approach. Instead, attention has turned to mouse genetics.

Hsa21 is orthologous to regions on mouse chromosomes 10 (Mmu10), Mmu16 and Mmu17. Using genome engineering, mouse strains have been constructed with duplications of each of these three regions, increasing their copy number from two to three (Lana-Elola et al., 2016; Li et al., 2007; Yu et al., 2010). We generated the Dp1Tyb mouse strain with an extra copy of 23 Mb of Mmu16, the largest of the Hsa21-orthologous regions, containing 145 coding genes, thereby modelling the trisomy of around 62% of Hsa21 (Lana-Elola et al., 2016). Analysis of Dp1Tyb mice has shown that they have many phenotypic features characteristic of DS in humans, including congenital heart defects, reduced bone density, and deficits in memory, locomotion, hearing and sleep (Chang et al., 2020; Lana-Elola et al., 2021, 2016; Thomas et al., 2020; Watson-Scales et al., 2018). Notably, we also found that Dp1Tyb mice have craniofacial phenotypes that recapitulate aspects of the human DS dysmorphology, including midfacial hypoplasia, brachycephaly and micrognathia (Toussaint et al., 2021). Similar findings have been reported for the Dp1Yey mouse strain, which has an additional copy of the same Mmu16 region (Li et al., 2007; Starbuck et al., 2014).

Here, we use morphometric analysis of a panel of mouse strains, each containing an extra copy of sub-regions of Mmu16, to map the location of genes causing the craniofacial phenotypes in Dp1Tyb mice. We discover that there must be at least four genes whose increased dosage contributes to the craniofacial dysmorphology and we show that one of these causative genes is *Dyrk1a*. We demonstrate that the most severe defects in Dp1Tyb skulls are in bones of neural crest (NC) origin and reveal aberrant mineralisation of the Dp1Tyb skull base synchondroses. Furthermore, we show that an increased dosage of *Dyrk1a* results in decreased NC cell proliferation and a decrease in the size of the NC-derived frontal bone primordia. Thus, DS craniofacial dysmorphology is caused by an increased dosage of *Dyrk1a* and at least three other genes, leading to reduced proliferation of NC cells that generate frontal and facial bones.

## RESULTS

### Genetic mapping identifies four loci causing the craniofacial phenotype in the Dp1Tyb model of DS

**Fig. 1.**
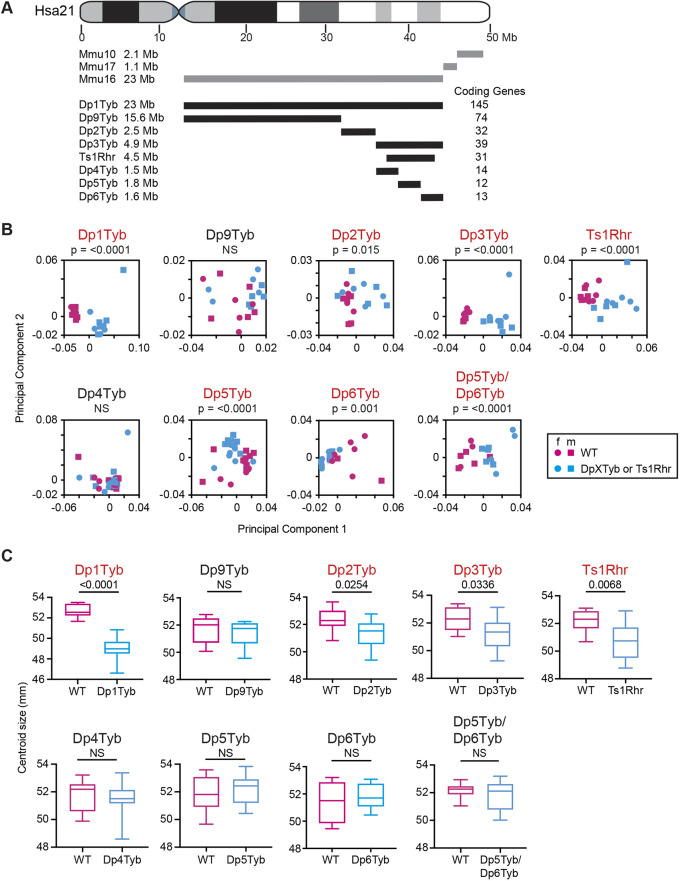
**Mapping genes that cause cranial dysmorphology in DS.** (A) Diagram of Hsa21 showing main cytogenic regions (rectangles of different shades) and the centromere (cyan). Regions of orthology to Mmu10, Mmu16 and Mmu17 are indicated by grey lines. Black lines represent duplicated regions in each mouse model, showing the number of coding genes within each region and their size in megabases (Mb). (B) PCA (first two components) of Procrustes aligned crania shapes determined using landmark-based morphometrics. Data from female (f) and male (m) mice are indicated separately. Percentage variance explained by each principal component is shown in Table S4. Statistical significance (*P*-values) of the difference between wild type and mutants was calculated using a multiple permutations test. (C) Centroid sizes of crania shown as box and whisker plots indicating the 25% and 75% centiles (box range), range of all data points (whiskers) and the median (line). Statistical significance was calculated using a two-tailed unpaired *t*-test. Data for Dp1Tyb mice are from our previous publication (Toussaint et al., 2021) where they were published under a CC-BY 4.0 license. *n*=9 or 10 for each genotype, except Dp4Tyb (15 wild type and 16 mutant), Dp5Tyb (17 wild type and 15 mutant) and Dp5Tyb/Dp6Tyb (seven wild type and 11 mutant). Mutant strains showing significant differences (*P*<0.05) are shown in red. NS, not significant (*P*>0.05).

To map the location of dosage-sensitive genes that cause the craniofacial dysmorphology of Dp1Tyb mice, we made use of a panel of seven mouse strains that have shorter genomic duplications contained within the Hsa21-orthologous region of Mmu16, thereby breaking up the 23 Mb, 145-gene region duplicated in Dp1Tyb mice into shorter segments (Fig. 1A) (Lana-Elola et al., 2016). We used micro-computed tomography (µCT) to generate 3D images of the skulls of 16-week-old mice from each of these seven strains (generally five mutant females and five mutant males), along with the same number of age- and sex-matched wild-type control mice. All mice were on a C57BL/6J background. To evaluate the shapes and sizes of the skulls, we carried out landmark-based morphometric analysis on the images, using 68 landmarks for the cranium and 17 landmarks for the mandible (Tables S1 and S2), comparing these with our previously published landmarking data from Dp1Tyb crania and mandibles (Toussaint et al., 2021). We evaluated shape changes using principal component analysis (PCA) and size changes by comparing centroid sizes. Our previous study had shown no sex difference in the shape of the cranium and only a subtle difference in the shape of the mandible (Toussaint et al., 2021). Importantly, for both cranium and mandible the effect of genotype was stronger than sex both for Dp1Tyb mice and the other strains, and thus both sexes were considered together.

The region duplicated in Dp1Tyb mice is broken down into three segments in Dp2Tyb, Dp3Tyb and Dp9Tyb mice (Fig. 1A). Similar to the changes seen in Dp1Tyb mice, the crania of Dp2Tyb and Dp3Tyb mice were significantly altered in shape and were smaller when compared with wild-type controls, whereas no change was seen in the crania of Dp9Tyb mice (Fig. 1B,C and Table S3). Ts1Rhr mice have a duplication that is entirely contained within the region duplicated in Dp3Tyb mice but is eight coding genes shorter (Fig. 1A) (Olson et al., 2004). Ts1Rhr mice showed altered shape and decreased size of the cranium similar to that seen in Dp3Tyb mice (Fig. 1B,C and Table S3). The Dp4Tyb, Dp5Tyb and Dp6Tyb strains each have a different short duplication that together cover the entire region duplicated in Dp3Tyb mice (Fig. 1A). No abnormality was seen in Dp4Tyb crania, while Dp5Tyb and Dp6Tyb crania were altered in shape but not in size (Fig. 1B,C, and Table S3). Thus, minimally, the cranial changes are caused by one or more genes in each of the non-overlapping Dp2Tyb, Dp5Tyb and Dp6Tyb regions.

**Fig. 2.**
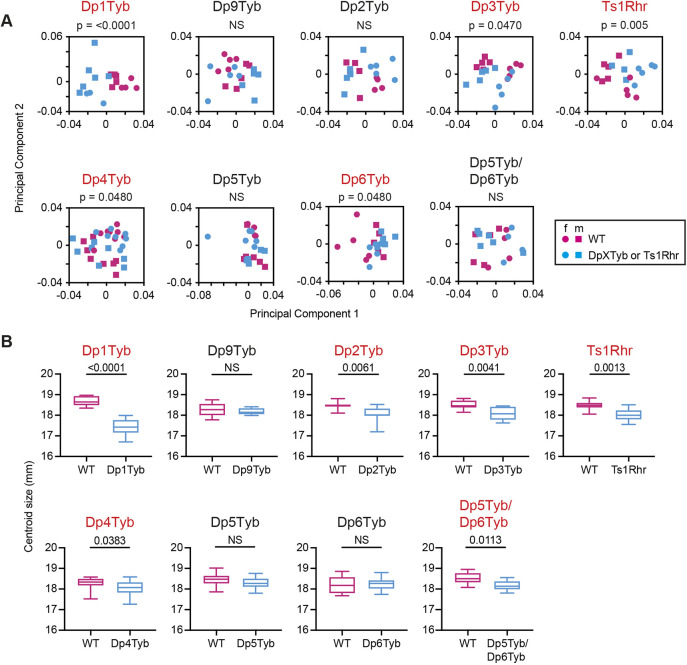
**Mapping genes that cause mandibular dysmorphology in DS.** (A) PCA of Procrustes aligned mandible shapes determined using landmark-based morphometrics. Data from female (f) and male (m) mice are indicated separately. Percentage variance explained by each principal component is shown in Table S4. Statistical significance was calculated using a multiple permutations test. (B) Centroid sizes of mandibles shown as box and whiskers plots indicating the 25% and 75% centiles (box range), range of all data points (whiskers) and the median (line). Statistical significance was calculated using a two-tailed unpaired *t*-test. Data for Dp1Tyb mice are from our previous publication (Toussaint et al., 2021) where they were published under a CC-BY 4.0 license. *n*=9 or 10 for each genotype, except Dp4Tyb and Dp5Tyb cohorts (*n*=16 for wild type and mutant). Mutant strains showing significant differences (*P*<0.05) are shown in red. NS, not significant (*P*>0.05).

Morphometric analysis of the mandibles showed that, similar to Dp1Tyb mice, they were decreased in size in Dp2Tyb and Dp3Tyb mice, but only Dp3Tyb mandibles were altered in shape, and again there were no changes in Dp9Tyb mandibles (Fig. 2A,B, Table S3). Once again, Ts1Rhr mandibles showed changed shape and decreased size, similar to Dp3Tyb mice. The mandibles of Dp4Tyb and Dp6Tyb mice showed a significant, albeit small, change in shape and Dp4Tyb mandibles were slightly smaller (Fig. 2A,B, Table S3). No changes were seen in Dp5Tyb mandibles. Thus, the genes causing the mandibular phenotype map to the Dp2Tyb, Dp4Tyb and Dp6Tyb regions, implying that there must be at least one causative gene in the Dp2Tyb region and at least two in the Dp3Tyb region (one in each of Dp4Tyb and Dp6Tyb).

**Fig. 3.**
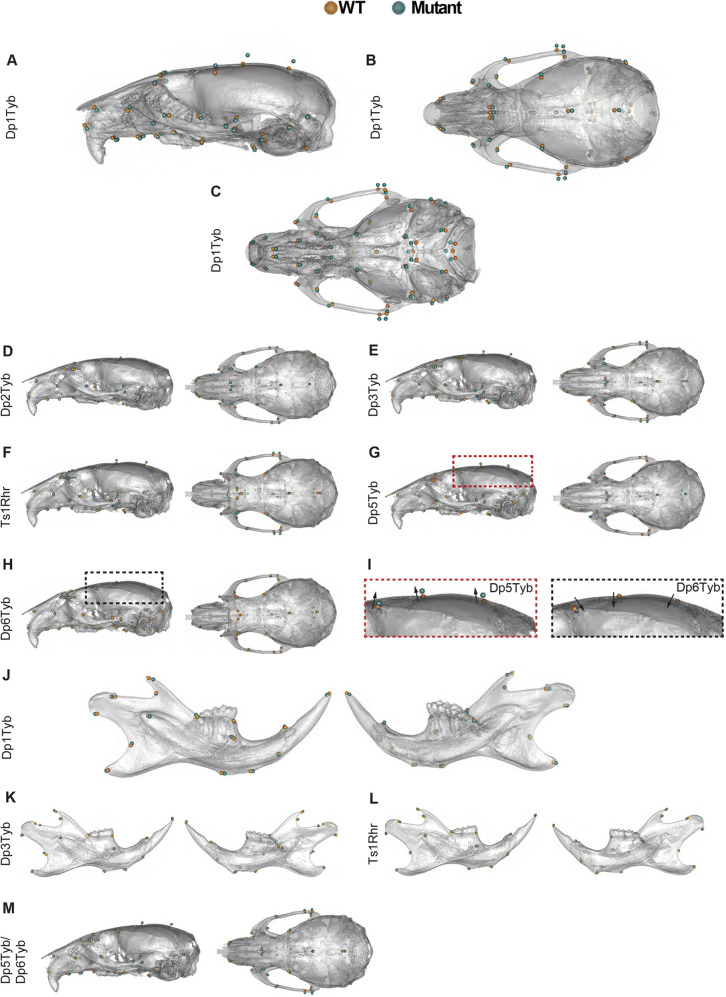
**Shape differences in DS mouse model skulls.** (A-C) Lateral, superior and inferior views of Dp1Tyb skulls adapted from Toussaint et al. (2021) where they were published under a CC-BY 4.0 license. (D-I) Mean landmark configurations of wild-type versus mutant crania with global size differences removed, showing lateral and superior views; (I) enlarged views of the regions outlined in G and H (left and right, respectively) showing the cranial vault of Dp5Tyb and Dp6Tyb mice. Arrows emphasise the direction of shape change. (J-L) Mean landmark configurations of wild-type versus mutant mandibles with global size differences removed, showing buccal and lingual views. (M) Mean landmark configurations of wild-type versus Dp5Tyb/Dp6Tyb crania with global size differences removed, showing lateral and superior views. Higher resolution views of these images are shown in Figs S1-S10.

The key aspects of the phenotype of Dp1Tyb crania are midfacial hypoplasia, cranial doming and occipital reduction. Together, these lead to a shortening of the anteroposterior axis, i.e. brachycephaly. These changes are very similar to those seen in humans with DS and are described in greater detail in our previous publication (Toussaint et al., 2021). To visualise these changes, we compared the locations of landmarks on Dp1Tyb crania with those in controls (Fig. 3A-C; see Fig. S1A-C for higher resolution images). These showed a posterior shift of anterior landmarks (cyan points in the left half of both Fig. 3A,B and Fig. S1A,B), widening of the skull (see cyan points on the zygomatic arches in the superior views of Fig. 3B, Fig. S1B), a doming of the skull (shown by the superior movement of the cyan points in the lateral view of Fig. 3A and Fig. S1A) and a contraction of the base of the skull (Fig. 3C and Fig. S1C). Similar changes were seen in Dp2Tyb, Dp3Tyb, Ts1Rhr and Dp5Tyb mice, although they were smaller in magnitude (Fig. 3D-G and Fig. S2-S5). In contrast, Dp6Tyb crania showed a ‘reverse’ phenotype with a more elongated midface, and a narrower and flatter skull (Fig. 3H,I and Fig. S6A,B). Similarly, comparison of the shapes of the mandibles showed that Dp3Tyb and Ts1Rhr had a contraction of the alveolar ramus and condylar process, which was similar to that seen in Dp1Tyb mice, albeit smaller in magnitude (Fig. 3J-L and Figs S7-S9).

We note that an earlier study reported that Ts1Rhr skulls have increased inter-landmark distances between the front and back of the cranium (Olson et al., 2004): the opposite of the observations we report here. The reason for this difference is unclear, but may be due to genetic background – we carried out all our studies on an inbred C57BL/6J background, whereas Olson et al. used mice on a B6xC3H segregating F2 background.

In the analysis of the three strains that break up the Dp3Tyb region, only Dp5Tyb and Dp6Tyb mice had cranial phenotypes, suggesting that the genes causing the Dp3Tyb phenotypes reside in these two regions. To test this, we generated Dp5Tyb/Dp6Tyb double mutant mice and analysed their skulls, as above. Compared with wild-type controls, the crania of Dp5Tyb/Dp6Tyb mice were altered in shape but not size (Fig. 1B,C and Table S3), whereas Dp5Tyb/Dp6Tyb mandibles were smaller but not changed in shape (Fig. 2A,B and Table S3). The shape phenotype in these mice was similar to the other strains in the mapping panel, with a shortened snout, doming of the skull and relative widening of the zygomatic arches (Fig. 3M and Fig. S10). Thus, combining the Dp5Tyb and Dp6Tyb mutations does not fully recapitulate the Dp3Tyb phenotype, implying that there must also be a genetic contribution from the Dp4Tyb region.

**Fig. 4.**
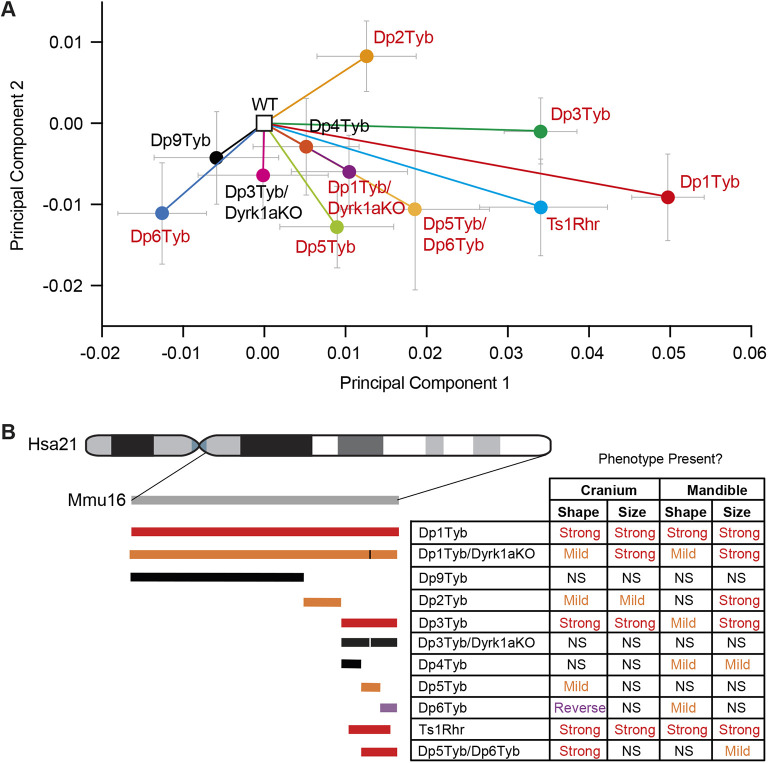
**Increased dosage of at least four genetic loci contribute to DS craniofacial dysmorphology.** (A) Normalised PCA plot of cranial shapes of the indicated mutant mouse strains. Plots have been normalised to place the mean PCA values for each wild-type cohort at the origin, with vectors indicating the mean PCA values (±s.e.m.) of each mutant strain. Mutant strains showing significant differences (*P*<0.05) compared with their wild-type controls are shown in red. Fig. S11 shows a similar PCA plot of all the wild-type cohorts before normalisation. (B) Summary of craniofacial phenotypes in the genetic mapping panel. Diagram of duplicated regions on the left as in Fig. 1A, with the addition of the Dp5Tyb/Dp6Tyb, Dp1Tyb/Dyrk1aKO and Dp3Tyb/Dyrk1aKO strains. Vertical lines indicate the location of the disrupted *Dyrk1a* gene. Duplicated regions are coloured to indicate the severity of the cranial shape phenotype: red, severe; orange, mild; purple, reversed phenotype in Dp6Tyb; black, no phenotype. Table shows whether cranial and mandibular phenotypes are present in the strains. NS, not significant (*P*>0.05).

To gain an overview of the shape changes across all the mouse strains in the mapping panel, we generated a PCA plot by directly comparing the landmark data for the crania from all mouse lines. The plot was normalised by placing the means of all the wild-type cohorts at the origin, allowing the direction and magnitude of the shape change of each mutant cohort to be directly compared (Fig. 4A). The DS-like cranial shape changes primarily separate the strains along principal component 1, with Dp1Tyb mice showing the largest change. Dp3Tyb and Ts1Rhr crania show a shape change in the same direction as Dp1Tyb, but smaller in magnitude. Leave-one-out cross-validation analysis shows that PCA is unable to robustly distinguish the two strains, implying that the additional eight genes that are in three copies in Dp3Tyb mice but not Ts1Rhr mice (Fig. S12) are not contributing significantly to phenotype. Dp5Tyb mice show a small shape change in the same direction as Dp3Tyb and Dp1Tyb mice, which is increased in the Dp5Tyb/Dp6Tyb double mutant but is still not as large as the change in Dp3Tyb mice, implying that there must also be causative genes in the Dp4Tyb region. Dp2Tyb mice also show a small phenotypic change in the direction of Dp1Tyb mice, suggesting that this region may contain one or more causative genes that, together with the causative genes in the Dp3Tyb region, give rise to the full Dp1Tyb phenotype. Finally, Dp6Tyb mice show a reverse phenotype in principal component 1, visible as a shape change in the opposite direction in many respects to the other strains. A summary of the phenotype mapping seen across all the strains is shown in Fig. 4B. Taken together, these results imply that the Dp1Tyb dysmorphology must be caused by the increased dosage of at least four genes, one in each of the regions duplicated in Dp2Tyb, Dp4Tyb, Dp5Tyb and Dp6Tyb mice (Fig. S12). Furthermore, the causative genes in the Dp4Tyb and Dp6Tyb regions most likely lie within the overlap with the region duplicated in Ts1Rhr, excluding the eight coding genes that are duplicated in Dp3Tyb but not Ts1Rhr.

### *Dyrk1a* is one of the genes required in three copies to cause craniofacial dysmorphology in Dp1Tyb mice

**Fig. 5.**
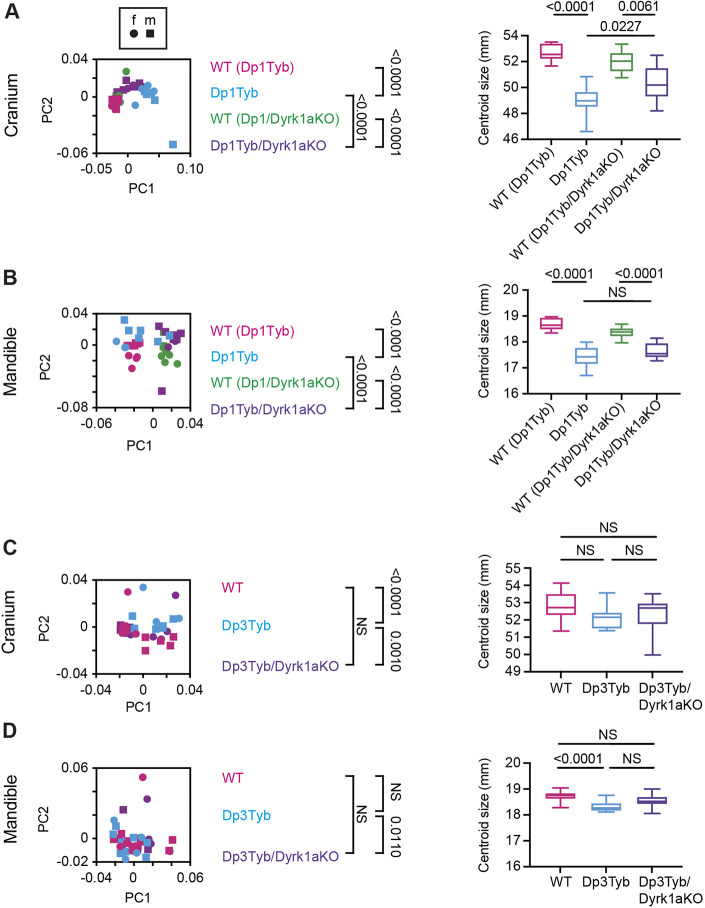
**Analysis of Dp1Tyb/Dyrk1aKO and Dp3Tyb/Dyrk1aKO mice.** (A-D) PCA of Procrustes aligned shapes determined using landmark-based morphometrics and centroid sizes of crania (A,C) and mandibles (B,D) of Dp1Tyb and Dp1Tyb/Dyrk1aKO mice, each with its own wild-type control (as indicated in parentheses) (A,B), and Dp3Tyb, Dp3Tyb/Dyrk1aKO and wild-type control mice (C,D). Centroid size shown as box and whiskers plots indicating the 25% and 75% centiles (box range), range of all data points (whiskers) and the median (line). Data from female (f) and male (m) mice are indicated separately. Statistical significance (*P*-values) was calculated using a multiple permutations test for PCA plots and a two-tailed unpaired *t*-test for centroid sizes, except for Dp1Tyb/Dyrk1aKO and Dp3Tyb/Dyrk1aKO centroid sizes, which were analysed by one-way ANOVA with Tukey's multiple comparisons test. Dp1Tyb/Dyrk1aKO crania and mandibles (*n*=9 or 10 for each genotype), and Dp3Tyb/Dyrk1aKO crania (17 wild type, nine Dp3Tyb, 13 Dp3TybDyrk1aKO) and mandibles (15 wild type, 12 Dp3Tyb and 13 Dp3TybDyrk1aKO). Dp1Tyb data in A and B are from Toussaint et al. (2021) where they were published under a CC-BY 4.0 license. NS, not significant (*P*>0.05).

The strain with the smallest duplicated region to show a craniofacial phenotype is Dp5Tyb, which contains an extra copy of just 12 coding genes. One of these genes, *Dyrk1a*, which encodes the DYRK1A serine/threonine protein kinase, has been shown to contribute to several DS phenotypes when present in three copies (Ahn et al., 2006; Altafaj et al., 2001; Brault et al., 2021; Duchon et al., 2021; Jiang et al., 2015; London et al., 2018; McElyea et al., 2016; Souchet et al., 2014; Thomazeau et al., 2014; Watson-Scales et al., 2018; Yin et al., 2017). Thus, we tested the requirement for three copies of *Dyrk1a* for the craniofacial phenotypes of Dp1Tyb and Dp3Tyb mice. We crossed mice with a loss-of-function *Dyrk1a* allele (*Dyrk1a*^+/−^) to Dp1Tyb and Dp3Tyb mice to generate Dp1Tyb/Dyrk1aKO and Dp3Tyb/Dyrk1aKO mice in which one of the three copies of *Dyrk1a* was inactivated, leaving two functional copies, while maintaining all other duplicated genes at three copies. Morphometric analysis of Dp1Tyb/Dyrk1aKO skulls showed that their crania and mandibles were altered in shape compared with wild-type controls, but were also significantly different from Dp1Tyb crania (Fig. 5A,B). Similarly, Dp1Tyb/Dyrk1aKO crania and mandibles were reduced in size, but the change was not as large as that seen in Dp1Tyb mice (Fig. 5A,B and Table S3). Analysis of Dp3Tyb/Dyrk1aKO crania and mandibles showed that they were not significantly different from wild-type controls in shape or size, unlike Dp3Tyb crania or mandibles (Fig. 5C,D, and Table S3). In the PCA of the cranial shapes of all strains, Dp1Tyb/Dyrk1aKO mice show a shape change in the same direction along principal component 1 as Dp1Tyb mice, but much smaller in magnitude (Fig. 4A). Thus, reducing *Dyrk1a* copy number from three to two partially rescues the Dp1Tyb and fully rescues the Dp3Tyb craniofacial dysmorphology, demonstrating that *Dyrk1a* is one of the causative genes (Fig. 4B, Fig. S12).

### Aberrant synchondroses in Dp1Tyb skulls

**Fig. 6.**
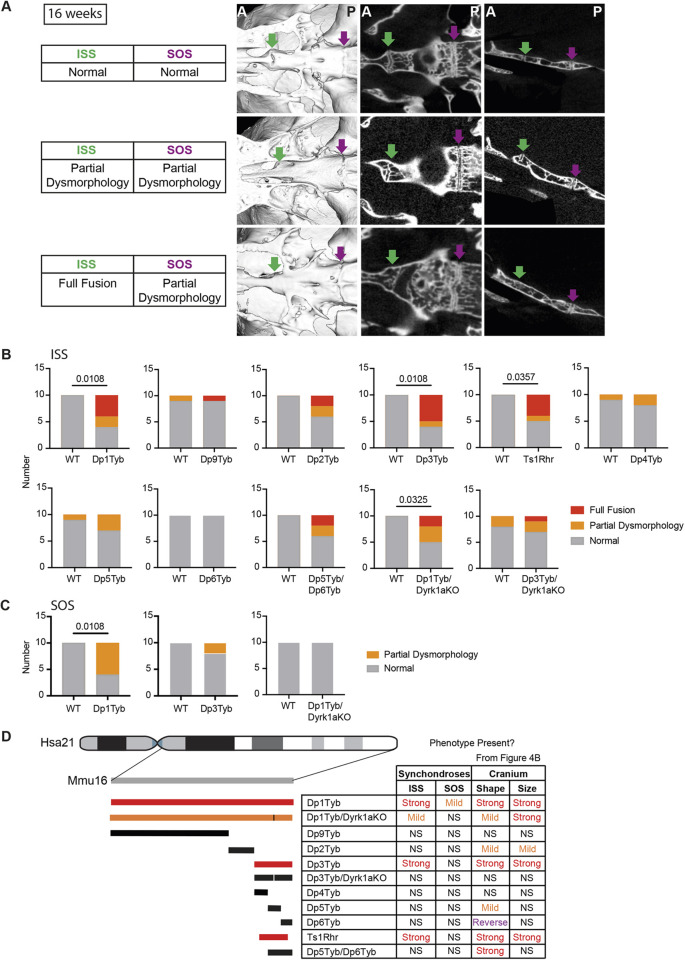
**DS mouse models have aberrant synchondroses.** (A) Inferior volumetric rendering (left), horizontal 2D slice (middle) and sagittal slice (right) of crania at 16 weeks of age, showing representative examples of synchondroses with normal, partially dysmorphic and fully fused phenotypes; green arrows, intersphenoid synchondroses (ISSs); magenta arrows, spheno-occipital synchondroses (SOSs). Image contrast was adjusted non-linearly using Photoshop in the slice images to remove signal from medium/soft tissue. Dysmorphic SOSs are shown in both the partial ISS dysmorphology and full ISS fusion images. A, anterior; P, posterior. (B,C) Incidence of fully fused, partially dysmorphic, and normal ISSs (B) and SOSs (C) in indicated mutant mouse strains. Only Dp1Tyb and Dp3Tyb are shown for SOS morphology because all other strains in the mapping panel showed no SOS dysmorphology. Statistical significance was calculated using Fisher's exact test, indicating significant differences (*P*<0.05). Where no *P*-value is shown, comparison of mutants with wild type is not significant (*P*>0.05). *n*=10 for each genotype. (D) Summary of synchondrosis phenotypes in the DS genetic mapping panel, as in Fig. 4B. Colours of duplicated regions indicate the severity of the ISS phenotype. Table shows whether ISS and SOS phenotypes are present in the strains, with the table of cranial phenotypes repeated from Fig. 4B to allow easier comparison. NS, not significant (*P*>0.05).

When studying the 16-week-old Dp1Tyb mice, we observed that cartilaginous growth points in the base of the cranium known as synchondroses were often dysmorphic. In mice, there are two midline synchondroses: the more anterior, intersphenoid synchondrosis (ISS) (Fig. 6A, green arrow) and the more posterior, spheno-occipital synchondrosis (SOS) (Fig. 6A, purple arrow). Using both volumetric reconstructions and digital slices of the base of the skull, we classified ISS morphology in each animal as normal, partially dysmorphic or fully fused, where no cartilaginous gap remained (Fig. 6A). Similarly, SOS morphology was classified as normal or partially dysmorphic. Dp1Tyb mice had a significantly increased frequency (>50%) of dysmorphic ISS and SOS (Fig. 6B,C). Analysis of synchondroses across the mapping panel showed that only Dp3Tyb and Ts1Rhr mice had significantly increased rates of dysmorphic ISS, although several other strains (Dp2Tyb, Dp5Tyb and Dp5Tyb/Dp6Tyb) showed evidence of dysmorphology that did not reach significance (Fig. 6B). Only Dp1Tyb mice had aberrant SOS (Fig. 6C). Disruption of one copy of *Dyrk1a* caused a reduction in the severity of ISS dysmorphology in Dp1Tyb mice and eliminated it in Dp3Tyb mice, and resulted in rescue of the SOS dysmorphology in Dp1Tyb mice (Fig. 6B,C). Thus, three copies of *Dyrk1a* are required for dysmorphic synchondroses. Taken together, the frequency of dysmorphic synchondroses tracked closely with cranial shape changes across the mapping panel, suggesting they may be caused by related pathological mechanisms (Fig. 6D).

### Developmental trajectory of craniofacial dysmorphology in Dp1Tyb mice

**Fig. 7.**
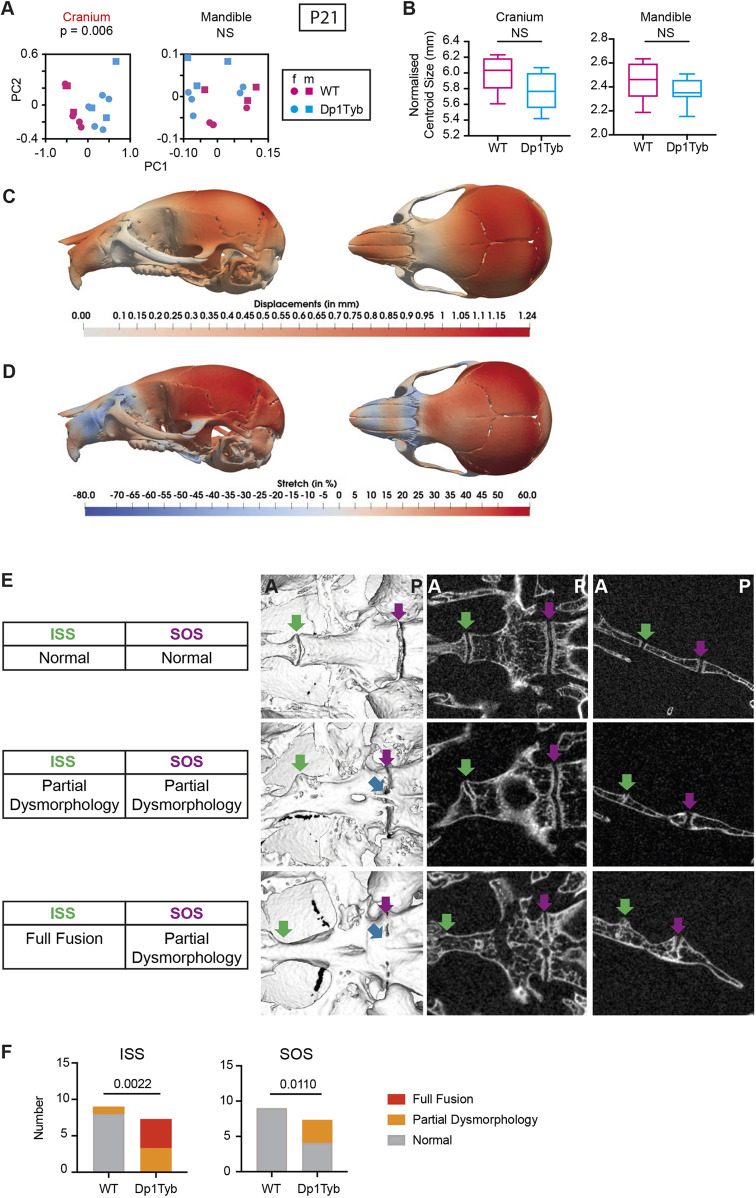
**Craniofacial dysmorphology in Dp1Tyb mice at P21.** (A,B) PCA of Procrustes aligned crania and mandible shapes determined using landmark-free morphometrics (A) and normalised centroid sizes of crania and mandibles (B) of P21 Dp1Tyb and wild-type control mice. Data from female (f) and male (m) mice are indicated separately. Centroid size data are shown as box and whiskers plots indicating the 25% and 75% centiles (box range), range of all data points (whiskers) and the median (line). (C,D) Heatmaps of displacement (C) and stretch (D) of Dp1Tyb P21 crania compared with wild-type controls, showing lateral and superior views. See also Movie 1. (E) Inferior volumetric rendering (left), horizontal 2D slice (middle) and sagittal slice (right) of crania at P21, showing representative examples of synchondroses with normal, partially dysmorphic and fully fused phenotypes; green arrows, intersphenoid synchondroses (ISSs); magenta arrows, spheno-occipital synchondroses (SOSs); blue arrows, mineralisation bridges. Dysmorphic SOSs are shown in both the partial ISS dysmorphology and full ISS fusion images. A, anterior; P, posterior. (F) Incidence of fully fused, partially dysmorphic, and normal ISSs and SOSs. Statistical significance (*P*-values) was calculated using a multiple permutations test (A), a two-tailed unpaired *t*-test (B) and Fisher's exact test (F). Wild type, *n*=9; Dp1Tyb, *n*=7. NS, not significant (*P*>0.05).

To gain a better mechanistic understanding of the origins of the craniofacial defects in Dp1Tyb mice, we analysed the skulls of young mice at 21 days post-partum (P21) by µCT and landmark-free morphometrics (Toussaint et al., 2021). We chose to use a landmark-free method for the analysis, because the changes at this age were more subtle than at 16 weeks, and thus more readily captured with landmark-free morphometrics compared with a landmark-based approach. The shape of the Dp1Tyb cranium was significantly different from wild-type controls, but the mandible shape was not affected and there were no significant differences in size of either cranium or mandible (Fig. 7A,B, Movie 1). Qualitatively, the cranial shape difference at this stage was similar to that observed at 16 weeks (Fig. 3A) (Toussaint et al., 2021). The snout was displaced towards the posterior and the calvaria were displaced laterally as seen in the displacement heat maps (Fig. 7C). This is further confirmed by the stretch heatmaps (Toussaint et al., 2021) which indicate that the snout is contracted and the calvaria are expanded as seen in the blue and red regions of Fig. 7D, respectively. We also examined the mid-line synchondroses in the cranial base. In WT control mice these were still patent, with no mineralisation (Fig. 7E,F), as expected based on previous analysis of C57BL/6J mice at P21 (Vora et al., 2016). In contrast, many Dp1Tyb mice had partially or fully fused ISS, with evidence of aberrant mineralisation bridges (Fig. 7E,F).

**Fig. 8.**
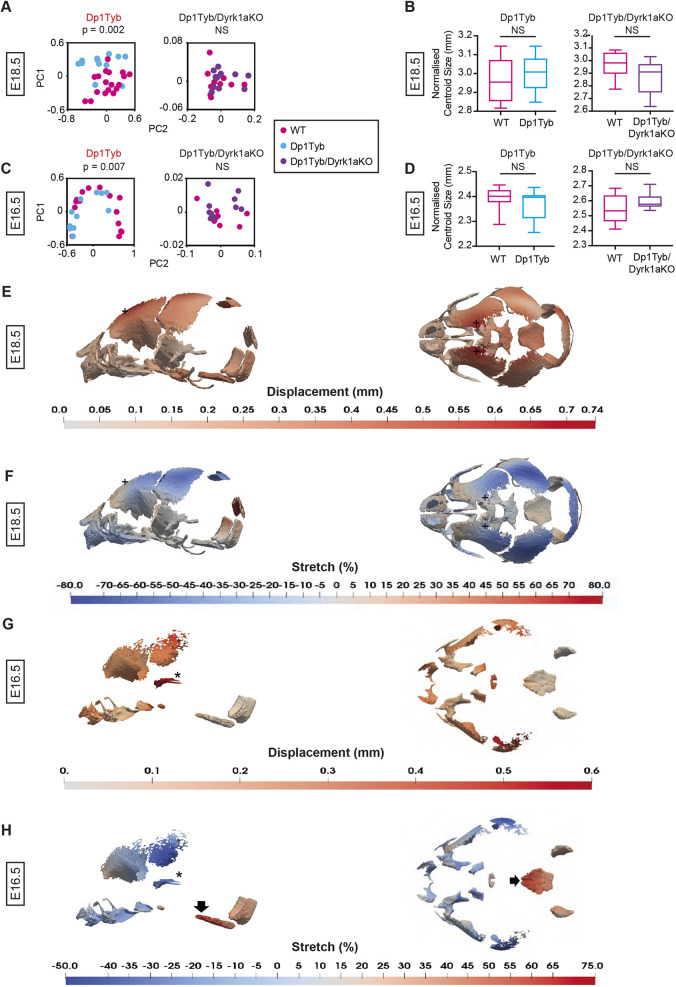
***Dyrk1a*-dependent craniofacial dysmorphology in Dp1Tyb embryos at E18.5 and E16.5.** (A-D) PCA of Procrustes aligned crania shapes determined using landmark-free morphometrics (A,C) and centroid sizes (B,D) of Dp1Tyb and Dp1Tyb/Dyrk1aKO embryos, and their respective wild-type controls at E18.5 (A,B) and E16.5 (C,D). Centroid size data shown as box and whiskers plots indicating the 25% and 75% centiles (box range), range of all data points (whiskers) and the median (line). Statistical significance (*P*-values) was calculated using a multiple permutations test for PCA plots and a two-tailed unpaired *t*-test for centroid sizes. At E18.5: wild type (Dp1Tyb), *n*=18; Dp1Tyb, *n*=12; wild type (Dp1Tyb/Dyrk1aKO), *n*=10; Dp1Tyb/Dyrk1aKO, *n*=12. At E16.5: wild type (Dp1Tyb), *n*=15; Dp1Tyb, *n*=11; wild type (Dp1Tyb/Dyrk1aKO), *n*=10; Dp1Tyb/Dyrk1aKO, *n*=11. (E-H) Heatmaps of displacement (E,G) and stretch (F,H) of Dp1Tyb crania compared with wild-type controls at E18.5 (E,F) and E16.5 (G,H), showing lateral and superior views. See also Movie 2. +, frontal bone (E,F); asterisk, zygomatic arch (G,H); black arrow, basioccipital bone (H). NS, not significant (*P*>0.05).

To extend this analysis further back in development, we studied the skulls of Dp1Tyb embryos at embryonic day 16.5 (E16.5) and E18.5 using µCT and landmark-free morphometrics (Toussaint et al., 2021), which allowed us to compare the developing cranial bones, which lack many of the usual landmarks. Crucially, it also allows analysis of local expansion or contraction (‘stretch’) rather than net displacement. We found that the crania of Dp1Tyb embryos at both E16.5 and E18.5 were significantly altered in shape (Fig. 8A,C, Movie 2), but there was no significant change in overall size (Fig. 8B,D). Examination of the shape changes showed that at E18.5 Dp1Tyb crania are flatter (Fig. 8E,F,+marks, Movie 2), in contrast to the domed phenotype seen at P21 and 16 weeks. However, like the postnatal stages, E18.5 Dp1Tyb crania had a contraction of the snout (Fig. 8F, blue region on the left-hand side). At E16.5 Dp1Tyb, crania showed a reduction in the size of the bones in the front half of the cranium (Fig. 8G, red regions, Fig. 8H, blue regions) and the zygomatic arch (Fig. 8G,H, asterisks). Strikingly, the E16.5 Dp1Tyb embryos also showed a relative expansion of the basioccipital and occipital condyle bones (Fig. 8H, black arrow and red region). Similar analysis of Dp1Tyb/Dyrk1aKO embryos showed that their crania were not significantly different from wild type in shape or size at either E16.5 or E18.5 (Fig. 8A-D). Thus, the changes in cranial shape of Dp1Tyb embryos are already visible at E16.5 and are dependent on three copies of *Dyrk1a*.

### An increased dosage of *Dyrk1a* reduces the size and differentiation of frontal bone primordia

**Fig. 9.**
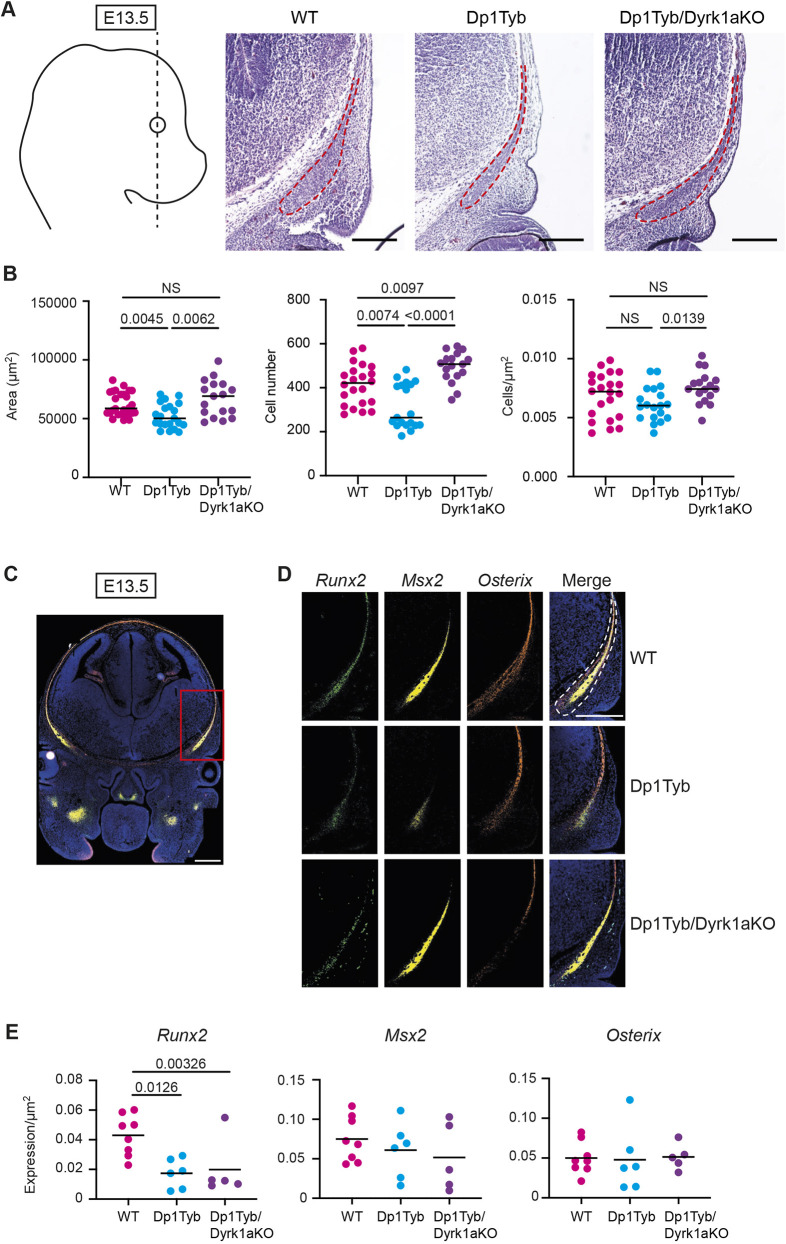
**Increased dosage of *Dyrk1a* causes decreased size of frontal bone primordia.** (A) Representative Hematoxylin and Eosin images of frontal bone primordia (dashed red line) of E13.5 embryos of the indicated genotypes sectioned coronally as shown by the dashed black line on diagram showing a lateral view of the embryo. (B) Area, cell number and cell density of coronal frontal primordia. Each dot is a different embryo; black line indicates the mean. (C,D) Representative RNAscope images of coronal sections through the heads of E13.5 embryos in the same plane as in A, showing hybridisation signals for *Runx2* (green), *Msx2* (yellow) and osterix (orange) mRNA expression, either merged (C) or separately and merged (D). The section in C is through a wild-type embryo showing the whole head and the red rectangle indicates the area shown at higher magnification in D. Sections in D are from embryos of the indicated genotypes. Dashed white line indicates the frontal bone primordium. Sections were stained with DAPI to visualise nuclei. (E) Mean of *Runx2*, *Msx2* and osterix mRNA expression in frontal bone primordia of E13.5 embryos of the indicated genotypes. Each dot is a result from an individual embryo; black line indicates the mean. Statistical significance was calculated using one-way ANOVA with Tukey's multiple comparisons test. NS, not significant (*P*>0.05). Scale bars: 200 µm.

Morphometric analysis of Dp1Tyb mice at several stages indicated that the most severely affected bones of the skull were those of NC origin, i.e. the frontal and facial bones and the ISS (Jiang et al., 2002; McBratney-Owen et al., 2008) with relatively modest abnormality in skull bones of mesodermal origin (the occipital and basioccipital) (Fig. 8H). The apparent size reduction in the NC-derived bones could be due to fewer cells being present or less µCT-detectable mineralisation, as the latter expands from the centres of the bone primordia towards their edges. To determine which of these was the case, we focussed on analysing development of one of these NC-derived structures: the frontal bones. Because a phenotype was already present in the frontal bones of Dp1Tyb embryos at early stages of ossification (E16.5), we assessed whether changes could be detected earlier in development by analysing the frontal bone primordia, which are mesenchymal condensations of NC origin, at E13.5 (Fig. 9A). The cells within these primordia will undergo differentiation into osteoblasts that produce mineralised bone. Histological analysis showed that frontal bone primordia of Dp1Tyb E13.5 embryos were smaller and had fewer cells (Fig. 9A,B). Notably, this defect was not seen in Dp1Tyb/Dyrk1aKO embryos (Fig. 9A,B). Thus, three copies of *Dyrk1a* are required for the decreased size and cellularity of frontal bone primordia in Dp1Tyb embryos.

Another mechanism that could lead to aberrant facial bones in Dp1Tyb embryos is altered ossification. To investigate this, we used RNAscope, a type of *in situ* hybridisation, on sections of the frontal bone primordia at E13.5 to quantify the expression of *Runx2*, *Msx2* and osterix (*Sp7*), genes that are crucial for the differentiation of mesenchymal cells into the osteogenic lineage (Han et al., 2007; Maeno et al., 2011; Matsubara et al., 2008; McGee-Lawrence et al., 2014; Nakashima et al., 2002) (Fig. 9C,D). The expression of the genes was normalised to area, to account for the reduced size of the primordia in Dp1Tyb embryos. We found that the expression of *Runx2*, but not *Msx2* or osterix, was reduced in Dp1Tyb primordia, suggesting that there may be ossification defects in the mutant embryos (Fig. 9E). Interestingly, reduced *Runx2* expression was still present in Dp1Tyb/Dyrk1aKO embryos; thus, this phenotype is caused by three copies of genes other than *Dyrk1a* (Fig. 9E).

### Increased dosage of *Dyrk1a* causes reduced proliferation of cranial neural crest cells

**Fig. 10.**
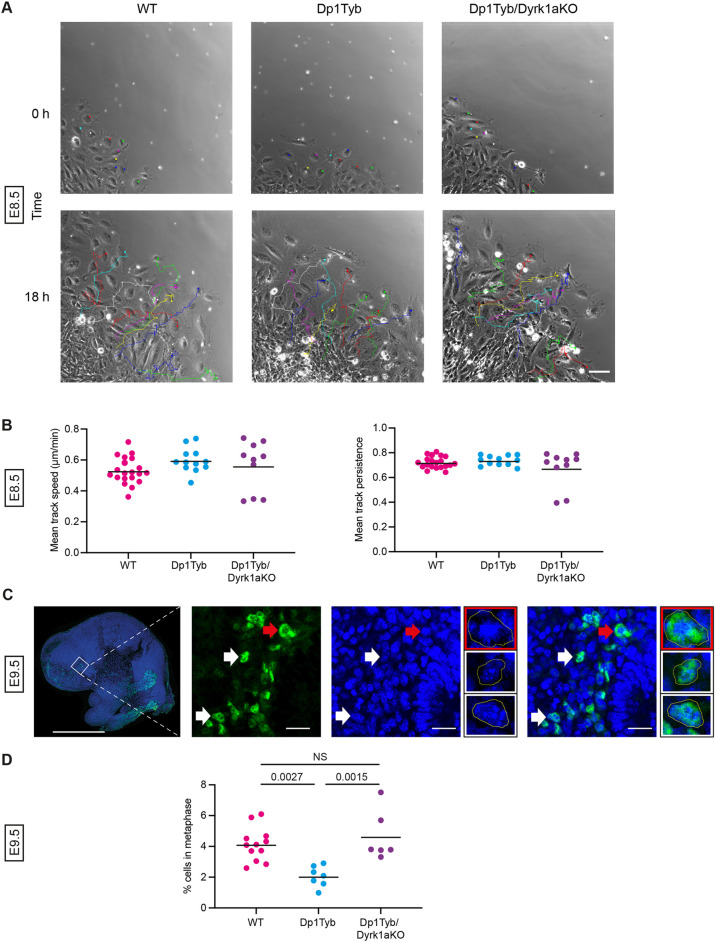
**Increased dosage of *Dyrk1a* causes reduced proliferation of cranial neural crest cells.** (A) Representative first and last frames of 18 h time lapse videos of tracked neural crest cells migrating out from a E8.5 neural tube explant of the indicated genotypes. Coloured dots in the first frame indicate cells whose migration is tracked and shown in the last frame. Scale bar: 100 µm. (B) Mean track speed and mean track persistence (straightness) of migrating neural crest cells from E8.5 neural tube explants of the indicated genotypes. Each dot is a result from an individual embryo; black line indicates the mean. (C) Representative low-magnification image (left) and higher magnification views (right three larger images) of a parasagittal section through an E9.5 mouse embryo head stained for SOX10 (green) and with DAPI (blue) in the facial prominence. White and red arrows indicate examples of SOX10^+^ cells that are not in metaphase or are in metaphase, respectively; these same cells are shown enlarged in the three smaller images, showing the SOX10^+^ cell in metaphase (red border) and two cells not in metaphase (white border). Yellow lines outline the cell. Scale bars: 1 mm (left image); 200 µm (right three larger images). (D) Percentage of SOX10^+^ cells that are in metaphase in the facial prominence of E9.5 embryos calculated from images such as those in C. Each dot is a result from an individual embryo; black line indicates the mean. Statistical significance was calculated using one-way ANOVA with Tukey's multiple comparisons test. NS, not significant (*P*>0.05).

The decrease in the size and cell number of Dp1Tyb frontal bone primordia could be due to defects in different processes. NC cells, the progenitors of this structure, undergo a key migratory phase early in their development and are also highly proliferative (Wu et al., 2017). A defect in either of these cellular behaviours could lead to the observed decrease in area and cellularity of the Dp1Tyb primordia; thus, we examined both processes. To measure migration, explants of E8.5 anterior neural tubes were cultured and individual migrating cells tracked for 18 h (Fig. 10A). We found that Dp1Tyb NC cells had no significant change in speed or persistence (straightness) of migration compared with wild-type cells (Fig. 10B), suggesting that early defects in NC migration are unlikely to account for the smaller frontal bone primordia in the mutant embryos.

Finally, we examined the proliferation of NC-derived cells in the facial prominence of E9.5 embryos by imaging these structures for SOX10 expression to identify NC-derived cells and DAPI to visualise cells in metaphase (Fig. 10C). We found that Dp1Tyb embryos had many fewer SOX10^+^ cells in metaphase compared with wild-type controls, implying that the Dp1Tyb mutation causes reduced proliferation of these cells, which may account for the reduced size and cellularity of the frontal bone primordia (Fig. 10D). Importantly, there was no proliferation deficit in Dp1Tyb/Dyrk1aKO embryos, demonstrating that increased dosage of *Dyrk1a* was required for reduced proliferation of NC-derived cells in the facial prominence.

Taken together, our results show that craniofacial dysmorphology in Dp1Tyb mice is caused by an increased dosage of at least four genes, one of which is *Dyrk1a*. Furthermore, we demonstrate that a third copy of *Dyrk1a* results in decreased proliferation of the NC-derived cells that give rise to frontal bone primordia. This may account for the altered sizes of the frontal bones and, ultimately, skull dysmorphology.

## DISCUSSION

DS is the most common cause of craniofacial dysmorphology in humans, yet the genetic and developmental mechanisms that underlie it are poorly understood. Taking advantage of the Dp1Tyb mouse model of DS with three copies of a 145-gene Hsa21-orthologous region of Mmu16 (Lana-Elola et al., 2016) and a DS-like craniofacial phenotype (Toussaint et al., 2021), we used genetic mapping to identify four shorter regions (A to D) of Mmu16 that each contain one or more dosage-sensitive genes required in three copies to cause the craniofacial dysmorphology (Fig. S12). Region A contains 32 coding genes duplicated in Dp2Tyb mice, region B contains 12 genes duplicated in Dp4Tyb but not Ts1Rhr mice, region C contains 12 genes duplicated in Dp5Tyb mice and region D contains 7 genes duplicated in Dp6Tyb but not Ts1Rhr mice.

We demonstrate here that *Dyrk1a* is one of the causative genes in region C, as reducing the copy number of *Dyrk1a* from three to two partially rescues the craniofacial phenotype of Dp1Tyb and fully rescues the phenotype of Dp3Tyb mice. DYRK1A is a protein kinase implicated in multiple DS phenotypes, which may be acting through several mechanisms (Ahn et al., 2006; Altafaj et al., 2001; Brault et al., 2021; Duchon et al., 2021; Jiang et al., 2015; London et al., 2018; McElyea et al., 2016; Souchet et al., 2014; Thomazeau et al., 2014; Watson-Scales et al., 2018; Yin et al., 2017). DYRK1A inhibits the function of NFAT transcription factors by phosphorylating them and thereby promoting their exit from the nucleus (Arron et al., 2006). As NFAT proteins regulate the differentiation of both osteoblasts and osteoclasts (Aliprantis et al., 2008; Koga et al., 2005; Lee et al., 2009; Winslow et al., 2006) and mice deficient in NFATC2 and NFATC4 have brachycephaly and mid-facial hypoplasia (Arron et al., 2006), increased dosage of *Dyrk1a* in Dp1Tyb mice may be promoting the craniofacial defects by inhibiting NFAT function and hence perturbing osteogenesis.

Alternatively, overexpression of *Dyrk1a* decreases cell proliferation (Liu et al., 2014; Yabut et al., 2010), while inhibition of DYRK1A leads to increased proliferation (Dirice et al., 2016; Shen et al., 2015). DYRK1A may affect proliferation by regulating Cyclin D1, a key regulator of the cell cycle (Sherr, 1995; Yang et al., 2006). DYRK1A phosphorylates Cyclin D1 on threonine 286 causing its degradation by the proteasome, thereby impairing progression from G1 to S phases of the cell cycle (Chen et al., 2013; Soppa et al., 2014). Thus, the increased dosage of *Dyrk1a* in Dp1Tyb mice may lead to decreased proliferation that could contribute to craniofacial defects. In support of this, we measured decreased proliferation of NC-derived cells in the frontal prominence of Dp1Tyb embryos, a phenotype that was dependent on three copies of *Dyrk1a*.

Other plausible causative genes include *Rcan1* in region A, *Morc3*, *Sim2* and *Ttc3* in region B and *Dscam* in region D. RCAN1 is an inhibitor of calcineurin, a phosphatase that dephosphorylates NFAT proteins, which promotes their nuclear entry. Thus, increased RCAN1 expression may cooperate with increased DYRK1A to inhibit NFAT function and thus impair osteogenesis (Arron et al., 2006). MORC3 is a nuclear protein that regulates osteoclast and osteoblast formation (Jadhav et al., 2016), hence an increased dosage of MORC3 in Dp1Tyb mice could also perturb osteogenesis. Mice deficient in the SIM2 transcription factor have craniofacial abnormalities, indicating that SIM2 plays an important role in craniofacial development (Shamblott et al., 2002). TTC3 targets AKT for ubiquitylation and reduces cell survival (Solzak et al., 2013; Suizu et al., 2009), so increased levels of TTC3 could result in reduced cell numbers, as we observed in the frontal bone primordia of Dp1Tyb embryos. Finally, DSCAM is a cell-adhesion molecule that has been proposed to act as a receptor for netrin 1, a laminin-related protein that inhibits osteoblast differentiation (Liu et al., 2009; Sato et al., 2017); thus, an increased dosage of DSCAM could affect osteoblastogenesis.

The craniofacial phenotype of Dp1Tyb mice is characterised by a shortening of the snout, contraction of the palate, doming of the skull, occipital reduction and micrognathia, and overall reduction in size (Toussaint et al., 2021). This dysmorphology remains largely consistent in models that break down the initial Dp1Tyb region, although the severity of the phenotype diminishes with decreasing size of duplication. The exception to this is the Dp6Tyb mouse strain, which showed a largely opposite phenotype with a dolichocephalic skull, i.e. longer and flatter than wild-type controls. Interestingly, combining the Dp5Tyb and Dp6Tyb increased dosage resulted in Dp1Tyb-like brachycephaly that was greater than that produced by Dp5Tyb alone, showing that increased dosage of the gene(s) causing the Dp6Tyb phenotype interact in a non-additive way with genes in the other regions.

One of the key characteristics of the craniofacial dysmorphology in Dp1Tyb mice is midfacial hypoplasia, as observed by the contraction of the snout and palate. This cranial phenotype correlated with aberrant synchondroses in Dp1Tyb mice, and other strains in the mapping panel, suggesting that these phenotypes may be linked. We found that both ISS and SOS show premature fusion in Dp1Tyb mice as early as P21, with evidence of mineralisation, which is normally absent in WT C57BL6/J mice at this age (Vora et al., 2016). Notably, premature fusion of synchondroses has also been reported in humans with DS at 7 months of age (Benda, 1940). Midfacial hypoplasia is commonly observed in other syndromes, such as Pfeiffer's and Crouzon's, and again strongly correlates with premature fusion of the synchondroses (Goldstein et al., 2014; Paliga et al., 2014; Tahiri et al., 2014). Furthermore, surgical immobilisation of the SOS in rabbits prevented growth of the skull along the anteroposterior axis (Rosenberg et al., 1997). This linkage between midfacial hypoplasia and premature fusion of synchondroses has led to two alternative hypotheses. First, premature fusion of the midline synchondroses may prevent anteroposterior growth, causing midfacial hypoplasia, brachycephaly and reduction in overall cranial size (Paliga et al., 2014; Tahiri et al., 2014). Alternatively, brachycephaly and midfacial hypoplasia may place the opposing edges of the synchondroses close together, increasing local tissue stiffness and thereby causing premature ossification of the structure (Mauney et al., 2004; Sathi et al., 2015).

The key structures affected by the dysmorphic phenotype of Dp1Tyb mice from E13.5 to 16 weeks of age are bones and structures of NC origin, such as the facial bones, frontal bones and the ISS (McBratney-Owen et al., 2008; Wu et al., 2017). We found that Dp1Tyb frontal bone primordia were reduced in size and cellularity at E13.5, and that the NC-derived cells of the frontal prominence, which will give rise to these primordia, had reduced proliferation at E9.5. All these phenotypes were dependent on three copies of *Dyrk1a*. We hypothesise that increased dosage of *Dyrk1a* leading to reduced proliferation of NC-derived cells results in smaller facial and frontal bones, and aberrant fusion of the synchondroses, which together lead to brachycephaly and midfacial hypoplasia. To extend this work in future studies, it will be interesting to analyse the expression of *Dyrk1a* in NC cells and their derivatives at multiple developmental stages, and to delete a single copy of *Dyrk1a* in Dp1Tyb NC cells to establish whether *Dyrk1a* plays a cell-autonomous role in the NC phenotype.

Earlier studies of the Ts65Dn mouse model of DS had postulated that craniofacial dysmorphology in these mice was also caused by increased dosage of *Dyrk1a* and a neural crest defect (McElyea et al., 2016; Roper et al., 2009). In one of these studies, explants of Ts65Dn neural crest cells showed defective migration (Roper et al., 2009), in contrast to our findings in Dp1Tyb mice. Ts65Dn mice not only have an extra copy of 131 Hsa21-orthologous genes from Mmu16, which corresponds to all the genes in the Dp2Tyb and Dp3Tyb regions and to some of the genes in the Dp9Tyb region, but also have an extra copy of 46 genes from Mmu17 that are not orthologous to Hsa21 (Duchon et al., 2011; Reinholdt et al., 2011). It is therefore not possible to know whether the phenotypes in this strain are derived from the increased dosage of Hsa21-orthologous genes or the latter non-orthologous genes, or a combination of both. This key genetic difference could account for our observation that neural crest migration was unaffected in Dp1Tyb embryos, in contrast to the results reported for Ts65Dn mice.

We observed that the frontal bone primordia of Dp1Tyb embryos expressed lower levels of *Runx2*, a key regulator of ossification (Komori et al., 1997; McGee-Lawrence et al., 2014). This deficit may also contribute to the craniofacial dysmorphology of Dp1Tyb mice. Since this reduction in *Runx2* expression does not depend on three copies of *Dyrk1a*, it may reflect a different pathological mechanism acting in parallel with DYRK1A-driven perturbations. Certainly, DYRK1A is not the whole story when it comes to the DS phenotype, and whether the other genes, which we have mapped to other regions, operate via DYRK1A-associated molecular pathways or more in parallel, interacting at the level of differentiation or morphogenesis, remains to be determined.

In conclusion, we have shown that craniofacial dysmorphology in the Dp1Tyb mouse model of DS, which resembles that seen in humans with DS, is caused by at least four genes, one of which is *Dyrk1a*. Furthermore, we show that increased dosage of *Dyrk1a* results in impaired proliferation of NC cells and subsequent reduced size and cellularity of frontal and facial bones, as well as abnormal mineralisation of midline synchondroses. Together, these perturbations give rise to the brachycephaly and midfacial hypoplasia that typifies the cranium in DS.

## MATERIALS AND METHODS

### Mice

C57BL/6J.129P2-Dp(16Lipi-Zbtb21)1TybEmcf (Dp1Tyb), C57BL/6J. 129P2-Dp(16Mis18a-Runx1)2TybEmcf (Dp2Tyb), C57BL/6J.129P2-Dp (16Mir802-Zbtb21)3TybEmcf (Dp3Tyb), C57BL/6J.129P2-Dp(16Mir802-Dscr3)4TybEmcf (Dp4Tyb), C57BL/6J.129P2-Dp(16Dyrk1a-B3galt5)5TybEmcf (Dp5Tyb), C57BL/6J.129P2-Dp(16Igsf5-Zbtb21)6TybEmcf (Dp6Tyb), C57BL/6J.129P2-Dp(16Lipi-Hunk)9TybEmcf (Dp9Tyb) and C57BL/6J.129S6-Dp(16Cbr1-Fam3b)1Rhr (Ts1Rhr) mice have been described previously (Lana-Elola et al., 2016; Olson et al., 2004). Dp1Tyb mice have been deposited with The Jackson Laboratory (strain 037183). All DpXTyb strains are available through the European Mouse Mutant Archive. Dp5Tyb and Dp6Tyb mice were inter-crossed to generate Dp5Tyb/Dp6Tyb double mutant mice. Dp1Tyb mice were crossed to mice heterozygous for the *Dyrk1a*^tm1Mla^ allele (*Dyrk1a*^+/−^, Dyrk1aKO) (Fotaki et al., 2002) to generate Dp1Tyb/*Dyrk1a*^+/+/−^ (Dp1Tyb/Dyrk1aKO) mice. As both Dp1Tyb and Dyrk1aKO mice are poor breeders, it was not possible to generate sufficient numbers of experimental mice with this cross. Thus, we identified mice in which a crossover had placed the Dyrk1aKO mutation *in cis* on the same chromosome as the Dp1Tyb mutation. The resulting Dp1Tyb/Dyrk1aKO mice were bred with wild-type mice, generating double mutants and wild-type animals in Mendelian ratios (1:1). Dp3Tyb mice were crossed to Dyrk1aKO mice to generate Dp3Tyb/*Dyrk1a*^+/+/−^ (Dp3Tyb/Dyrk1aKO) mice. All mice were bred at the MRC Harwell Institute (UK), except Dp3Tyb/Dyrk1aKO mice, which were bred at The Francis Crick Institute (London, UK). All mice were backcrossed to C57BL/6J for at least 10 generations. However, as the DpXTyb strains and the Dyrk1aKO strain were all generated on a 129P2 mouse genetic background, a small region of the 129P2 mouse genome will inevitably persist around the position of these mutations and may affect the phenotypes being studied. All animal work was approved by the Ethical Review panel of the Francis Crick Institute and was carried out under Project Licences granted by the UK Home Office. Numbers of protein-coding genes in different mouse strains were determined using the Biomart function in Ensembl on mouse genome assembly GRCm39, filtering for protein-coding genes, excluding three genes: ENSMUSG00000116933, which is a partial transcript for *Atp5o* (ENSMUSG00000022956); *Gm49711*, which is an alternatively spliced form of *Mrps6*; and *Gm49948*, which is a fusion transcript of exons from *Igsf5* and *Pcp4*. The numbers of coding genes listed in Fig. 1A are slightly different from those reported in our original publication of Dp1Tyb mice and the related strains (Lana-Elola et al., 2016), due to changes in the annotation of the mouse genome.

### Embryo collection

The day a vaginal plug was found was designated embryonic day 0.5 (E.5). Embryos were collected at E8.5, E9.5, E13.5, E16.5 and E18.5. Embryos were dissected out and those younger than E14.5 were immediately placed into ice-cold PBS. Embryos older than E14.5 were decapitated. For Haematoxylin and Eosin staining and X-ray μCT analysis, heads were fixed in 4% PFA at 4°C overnight. For RNAscope analysis, heads were fixed in 10% neutral buffered formalin (NBF) for 16-32 h at room temperature. For immunofluorescence assays, whole embryos at E9.5 were fixed for 1 h at room temperature.

### X-ray microcomputed tomography

For analysis of post-natal mice (16 weeks of age or P21), we generally used 10 mutant animals of each strain (five female and five male) and 10 wild-type control age- and sex-matched mice from the same litters. Mice were killed via cervical dislocation. Heads were dissected from bodies and fixed for 24 h in 4% paraformaldehyde (PFA). Heads of 16-week-old and P21 mice were rinsed in PBS for 2×1 h and then scanned in a SCANCO μCT 50 at 20 μm resolution, at 70 kV and with 500 3 s projections. Heads of embryonic mice were rinsed in PBS for 2×1 h and then scanned in a SCANCO μCT 50 at 9 μm resolution, at 90 kV and with 1500 3 s projections.

### Landmark-based morphometric analysis

Sixty-eight three-dimensional landmarks for the crania and 17 landmarks for the mandible were used as previously defined (Hallgrímsson et al., 2007; Toussaint et al., 2021). Images were processed to isolate bone structures and the mandible was manually separated from the rest of the skull to facilitate viewing. Landmarks were placed manually on a 3D volumetric reconstruction of the μCT images using the Microview (Parallax innovations) software; the same operator placed landmarks for all strains and was always blinded to genotype. Positioning of landmarks was checked using orthogonal planar views of the scanned subject. The same landmark sets were used for all mice. Landmark coordinate data are collated in Tables S1 and S2. PCA was conducted in MorphoJ (Klingenberg, 2011) to visualise group shape separation. Percentage variance accounted for by each principal component is shown in Table S4. Statistical significance of shape differences was quantified by using the Procrustes Distance Multiple Permutations test at 1000 iterations in MorphoJ. Centroid size, defined as the square root of the sum of the squared distances of all landmarks from the centroid (the average *x*, *y*, *z* coordinate for each landmark dataset), was calculated using MorphoJ. Statistical significance of size differences was calculated using a two-tailed unpaired *t*-test, where two groups were being compared, or one-way ANOVA with Tukey's multiple comparisons test, where three or more groups were being compared.

To compare the shape changes between mutant mouse strains, PCA was carried out on the crania from all the mouse strains together. The resulting PCA values for the cranial shapes of each mutant strain were normalised to place the mean PCA values for each wild-type cohort at the origin of the PCA plot (Fig. 4A). Fig. S11 shows a PCA plot from this analysis of all the wild-type cohorts before normalisation.

### Landmark-free morphometric analysis

Landmark-free analysis of P21, E18.5 and E16.5 mice was conducted as previously described (Toussaint et al., 2021). In brief, thresholding was applied to the µCT images, extracting and generating a binary mask of the skull structures. Mandibles were separated from the rest of the cranium using segmentation based on bone density. Meshes of both the mandible and cranium were generated and aligned using Procrustes-based superimposition. Aligned meshes were subjected to atlasing using Deformetrica. Finally, outputs of atlas construction were analysed by PCA and a stratified k-fold cross validation test performed on the output PCA data. Significance of the classification score was then tested using a multiple permutations test at 1000 iterations. Normalised centroid size, defined as the square root of the sum of the squared distances of all landmarks from the centroid (the average *x*, *y*, *z* coordinate for each landmark dataset) divided by the number of mesh vertices, was calculated using MorphoJ. Statistical significance of size differences was calculated using a two-tailed unpaired *t*-test.

### Paraffin wax embedding

Following fixation, embryos were washed in PBS for 3×30 min, then dehydrated through a series of ethanol solutions of 30%, 50%, 70%, 80%, 90% and 100% for 1 h each at room temperature on a nutator and subsequently placed in cassettes. Using a Leica ASP300 tissue processor, embryos were place in xylene for 3×1 h and Ultraplast Wax for 3×1 h at 63°C. Following this, heads were placed in metal moulds in the desired orientation, embedded in paraffin wax and left to cool on an ice block. Once the wax had solidified, they were stored at 4°C until required.

### Microtome sectioning

Embryos embedded in paraffin blocks were trimmed down to an appropriate size using a razor blade and then sectioned on a Leica RM2245 microtome with disposable Leica microtome blades at a 10 μm. Serial sections were placed into a 42°C deionised water bath to help with mounting onto Superfrost Plus slides. Slides were dried overnight on a heat block. For RNAscope, sections were cut to 5 μm and dried overnight at room temperature.

### Histology

Sections (10 μm) were obtained and stained with Haematoxylin and Eosin for visualisation of the frontal bone mesenchymal condensation. For immunofluorescence, antigen retrieval was conducted by incubating whole E9.5 mouse embryos in Tris-EDTA buffer [10 mM Tris Base, 1 mM EDTA, 0.05% Tween 20 (pH 8.0)] for 1 h. The embryos were washed in PBS and underwent permeabilisation in 0.5% PBST for 30 min. They were washed again and subsequently blocked with 20% normal goat serum (NGS) for 1 h and incubated overnight at 4°C with rabbit polyclonal antibody against Sox10 (Proteintech, 10422-1-AP) in 20% NGS. Sections were washed with PBS, blocked in 20% NGS for 1 h and incubated with goat anti-rabbit IgG Alexa488 and DAPI overnight at 4°C. Finally, sections were washed with PBS and mounted with ProLong Diamond Antifade Mountant (Thermo Fisher Scientific).

### Neural crest cell migration assay

Primary mouse cranial neural crest explant cultures were performed as previously described (Gonzalez Malagon et al., 2019). Embryos were harvested at E8.5 and immediately placed into ice-cold PBS. The anterior neural plate border region was dissected out and plated onto a glass-bottomed, 24-well tissue culture plate (Ibidi) coated with 1 µg/ml fibronectin. Explants were cultured overnight at 37°C and 5% CO_2_ in neural crest media containing Dulbecco's modified Eagle's medium, 15% fetal bovine serum, 0.1 mM minimum essential medium nonessential amino acids, 1 mM sodium pyruvate, 55 µM β-mercaptoethanol, 100 units/ml penicillin, 100 units/ml streptomycin and 2 mM L-Glutamine, conditioned by growth-inhibited STO feeder cells. Phase-contrast imaging was carried out using an IX 81 microscope (Olympus), with a Cascade II 512B camera (Photometrics),and a 10x UPlanFL NA1.45 objective lens for 18 h every 5 min. Cells were tracked manually by following their nuclear position using the Manual Tracking plug-in (Fiji). Cell tracks were imported into Wolfram Mathematica to analyse mean track speed and mean track persistence using the Chemotaxis Analysis Notebook v1.5β (created by G. Dunn, King's College London, UK). Mean track speed is the average cell speed over the whole track length. Persistence was defined as the ratio of the total displacement of the cell to the total distance travelled over a time interval of 20 min. Mean track persistence is the average persistence over multiple time intervals (Law et al., 2013). The metrics were analysed using Prism 8 (GraphPad) to generate graphs and to calculate statistical significance using Tukey's multiple comparisons test.

### RNAscope *in situ* hybridisation

Multiplex RNAscope *in situ* hybridisation was conducted using the RNAscope multiplex V2 reagent kit (ACD) and probes against *Runx2* (414021), osterix (403401-C2) and *Msx2* (421851, all from ACD). Slides were imaged as a tile scan using a Vectra Polaris Automated Quantitative Pathology Imaging System (Akoya Biosciences) at 20× magnification. Regions of interest were selected using the whole slide scan viewer Phenochart (Akoya Biosciences) for unmixing. Tiles of the image were spectrally unmixed using inForm Tissue Analysis Software (Akoya Biosciences), stitched together in Qupath and the frontal bone mesenchymal condensation was outlined. The total area of fluorescence for each gene in this area was calculated using the cell detection tool and divided by the total area to give expression per μm^2^. The average measurements for each slide were analysed using one-way ANOVA with Tukey's multiple comparisons test to calculate statistical significance.

## Supplementary Material

10.1242/develop.201077_sup1 Supplementary information
