# Supplementary material for: Craniofacial dysmorphology in Down syndrome is caused by increased dosage of Dyrk1a and at least three other genes
Source: Development. 2023 Apr 26;150(8):dev201077. doi: 10.1242/dev.201077 (PMC10163349; doi:10.1242/dev.201077)
Supplement: Supplementary information [file develop-150-201077-s1.pdf]

Figure S1

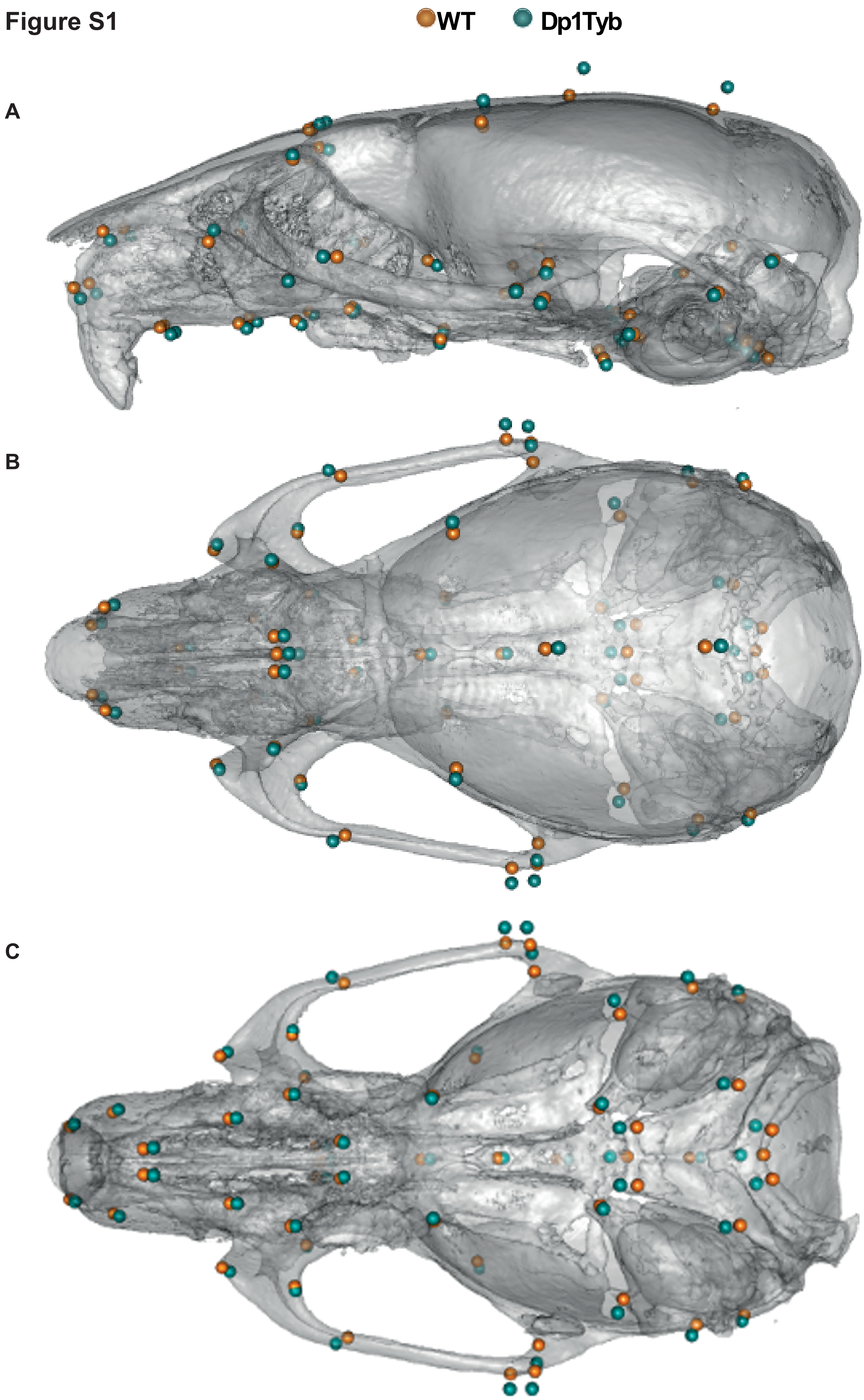

Fig. S1. Shape differences in Dp1Tyb mouse crania.
Mean landmark configurations of WT versus Dp1Tyb crania with global size differences removed, showing lateral (A), superior (B) and inferior (C) views. Data are from our previous publication (Toussaint et al., 2021) where they were published under a CC-BY 4.0 license.

Figure S2

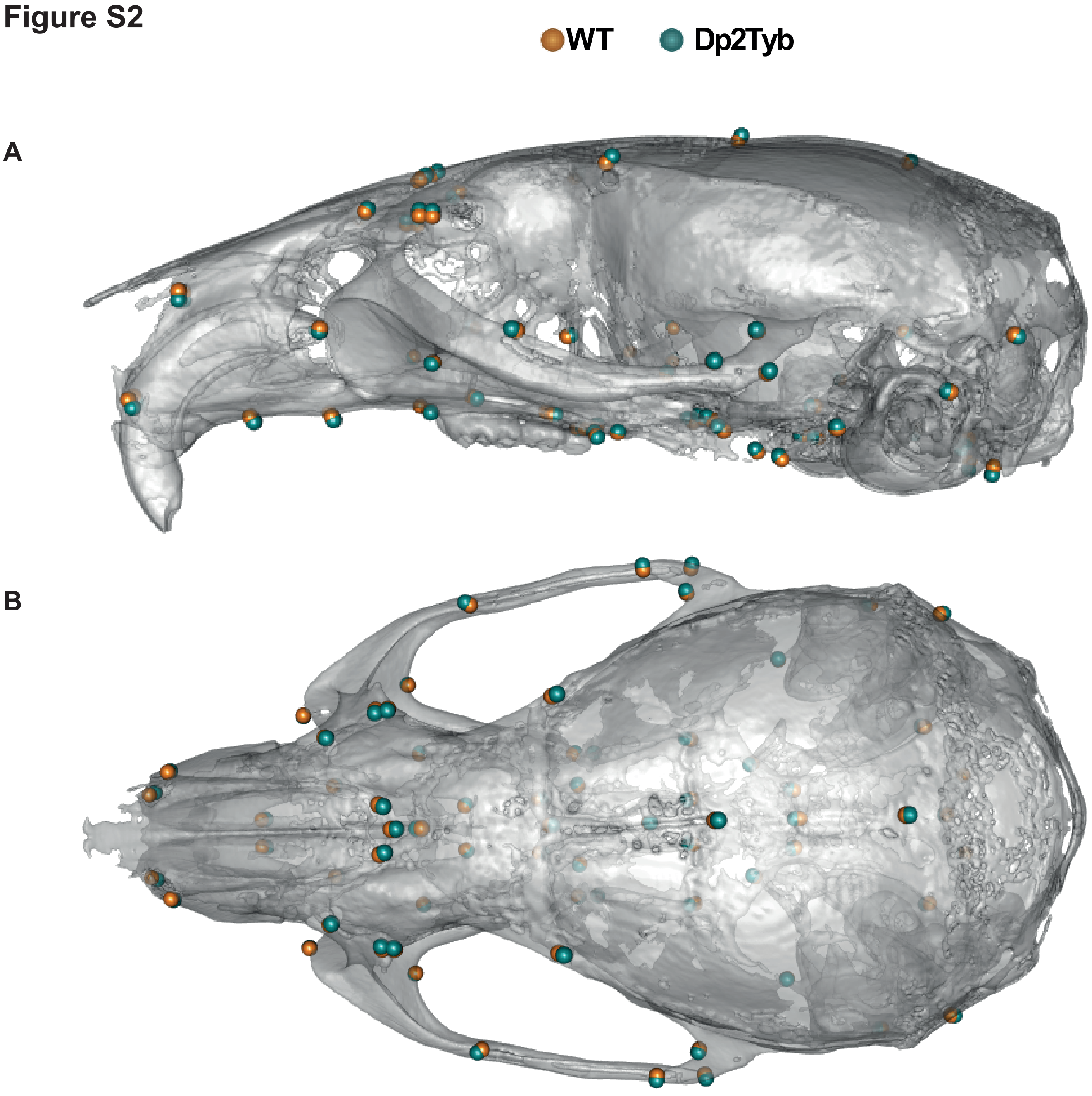

Fig. S2. Shape differences in Dp2Tyb mouse crania.
Mean landmark configurations of WT versus Dp2Tyb crania with global size differences removed, showing lateral (A) and superior (B) views.

Figure S3

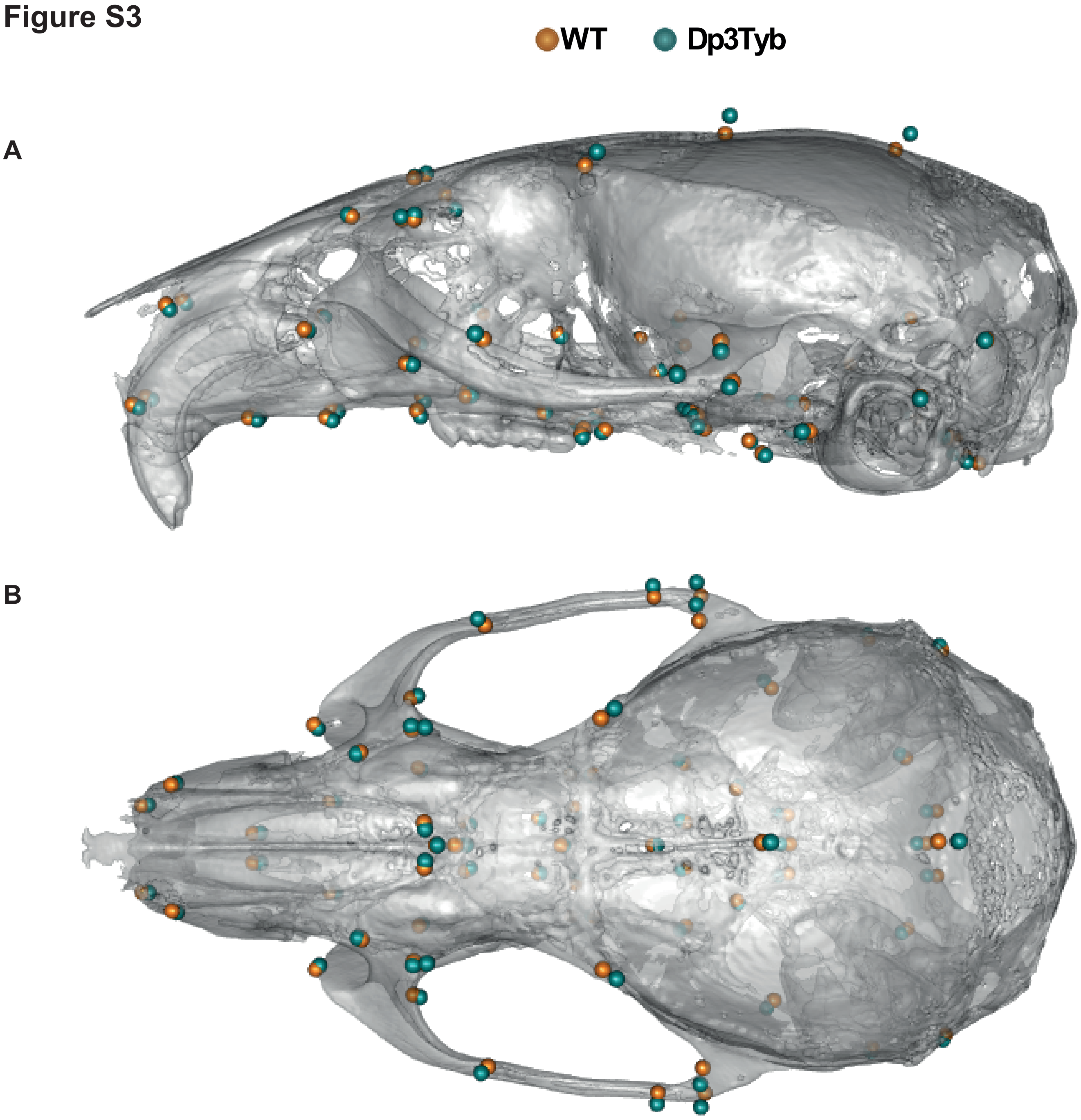

Fig. S3. Shape differences in Dp3Tyb mouse crania.
Mean landmark configurations of WT versus Dp3Tyb crania with global size differences removed, showing lateral (A) and superior (B) views.

Figure S4

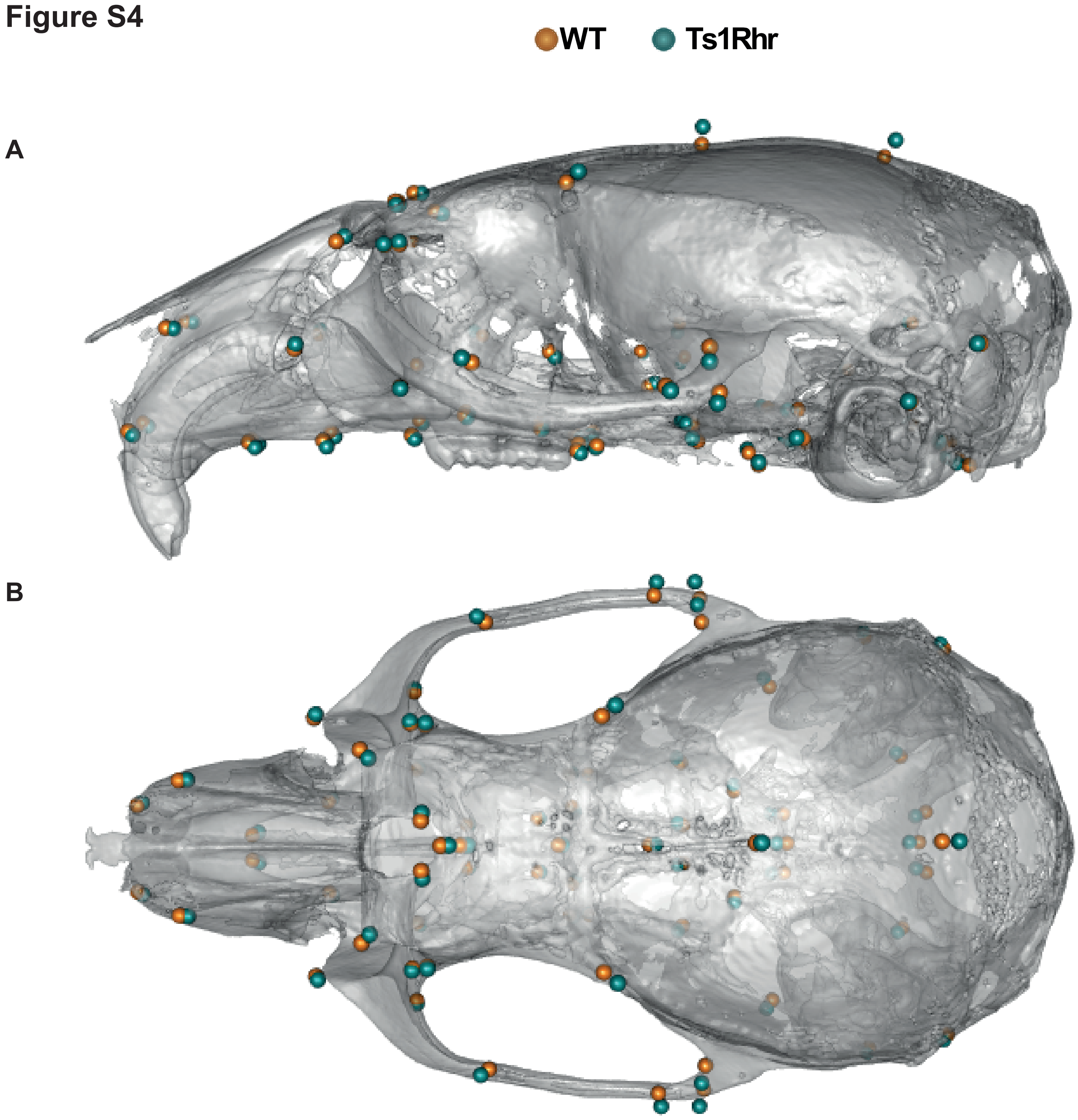

Fig. S4. Shape differences in Ts1Rhr mouse crania.
Mean landmark configurations of WT versus Ts1Rhr crania with global size differences removed, showing lateral (A) and superior (B) views.

Development • Supplementary information

Figure S5

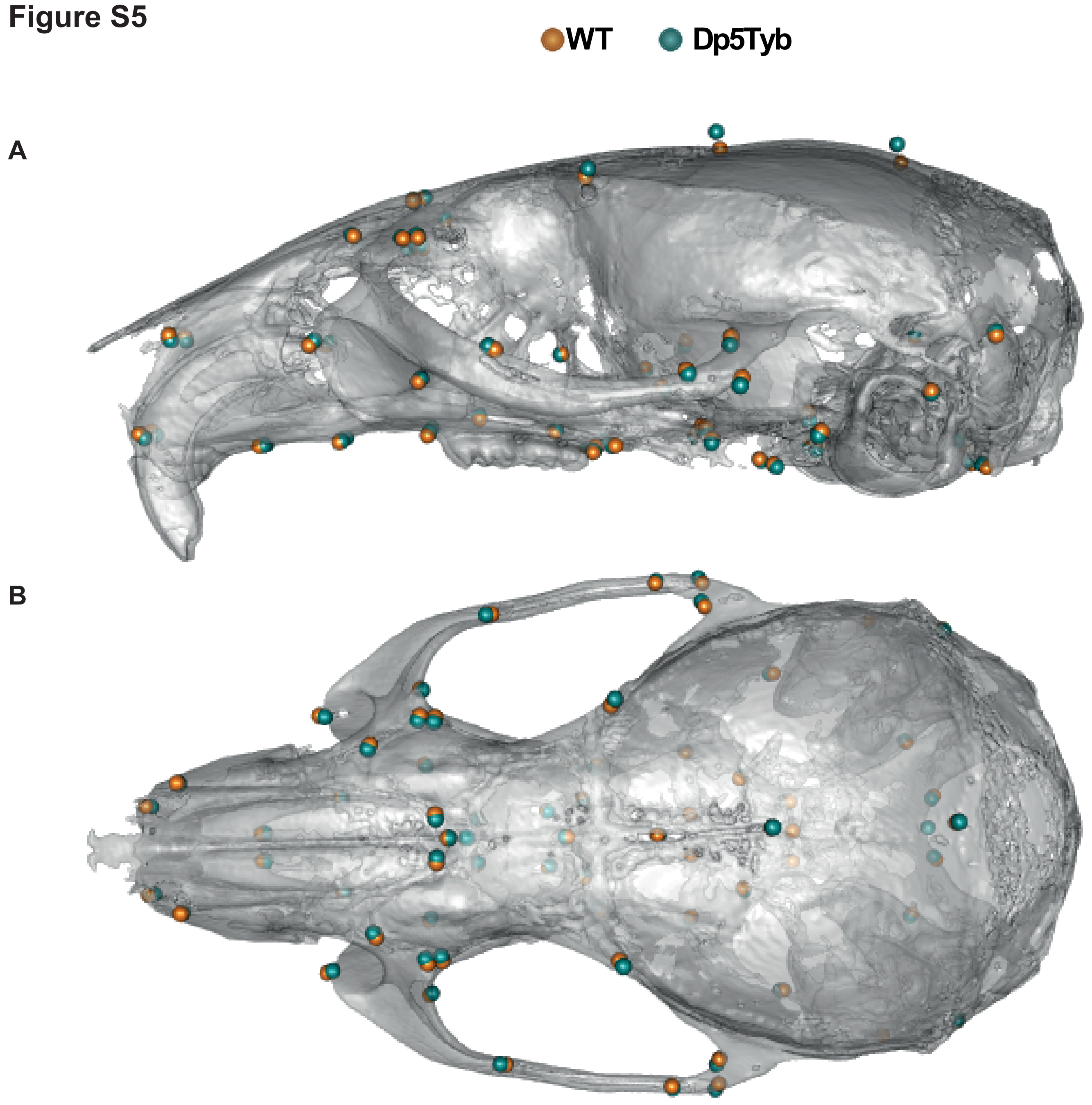

Fig. S5. Shape differences in Dp5Tyb mouse crania.
Mean landmark configurations of WT versus Dp5Tyb crania with global size differences removed, showing lateral (A) and superior (B) views.

Development • Supplementary information

Figure S6

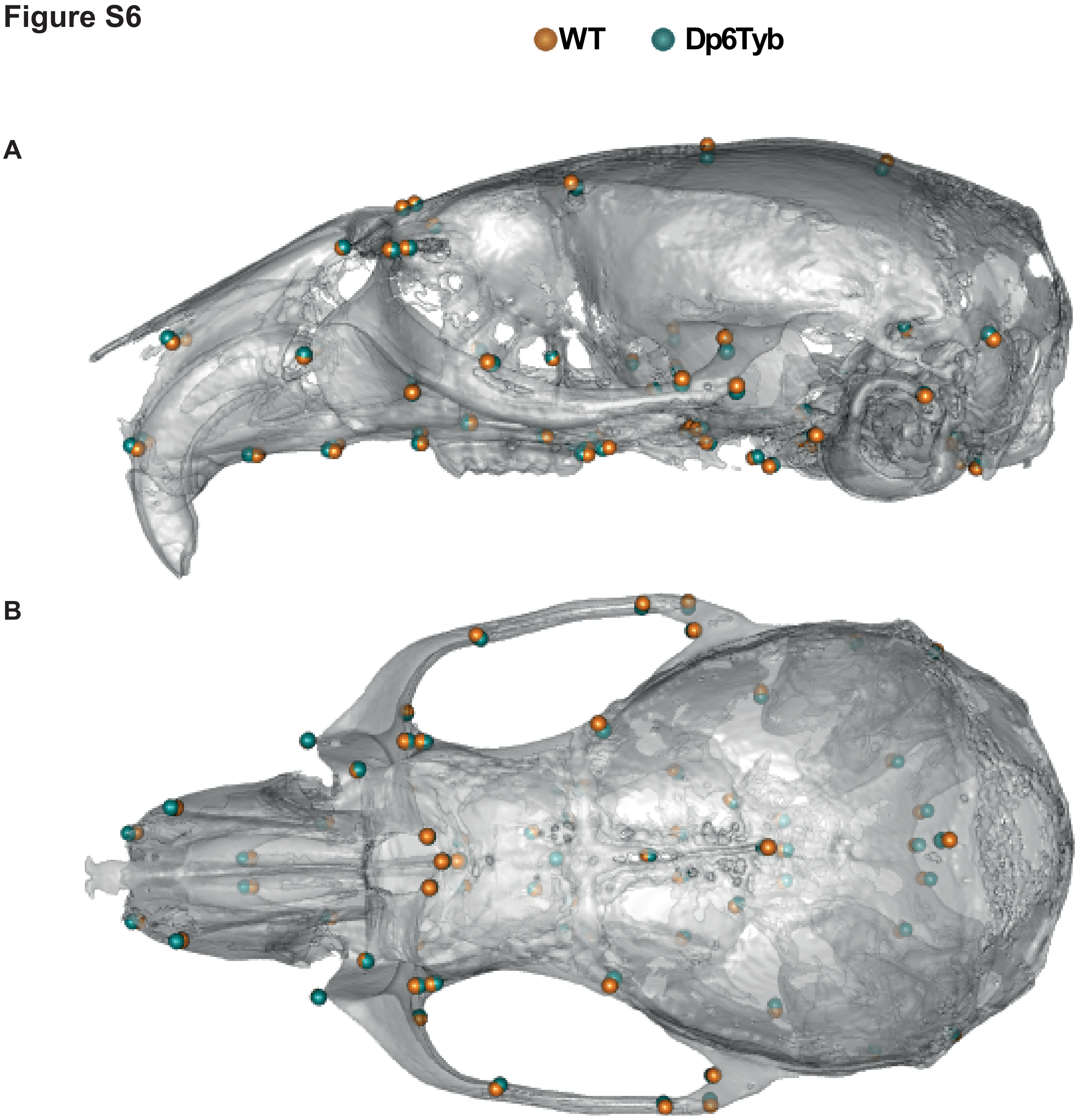

Fig. S6. Shape differences in Dp6Tyb mouse crania.
Mean landmark configurations of WT versus Dp6Tyb crania with global size differences removed, showing lateral (A) and superior (B) views.

Figure S7

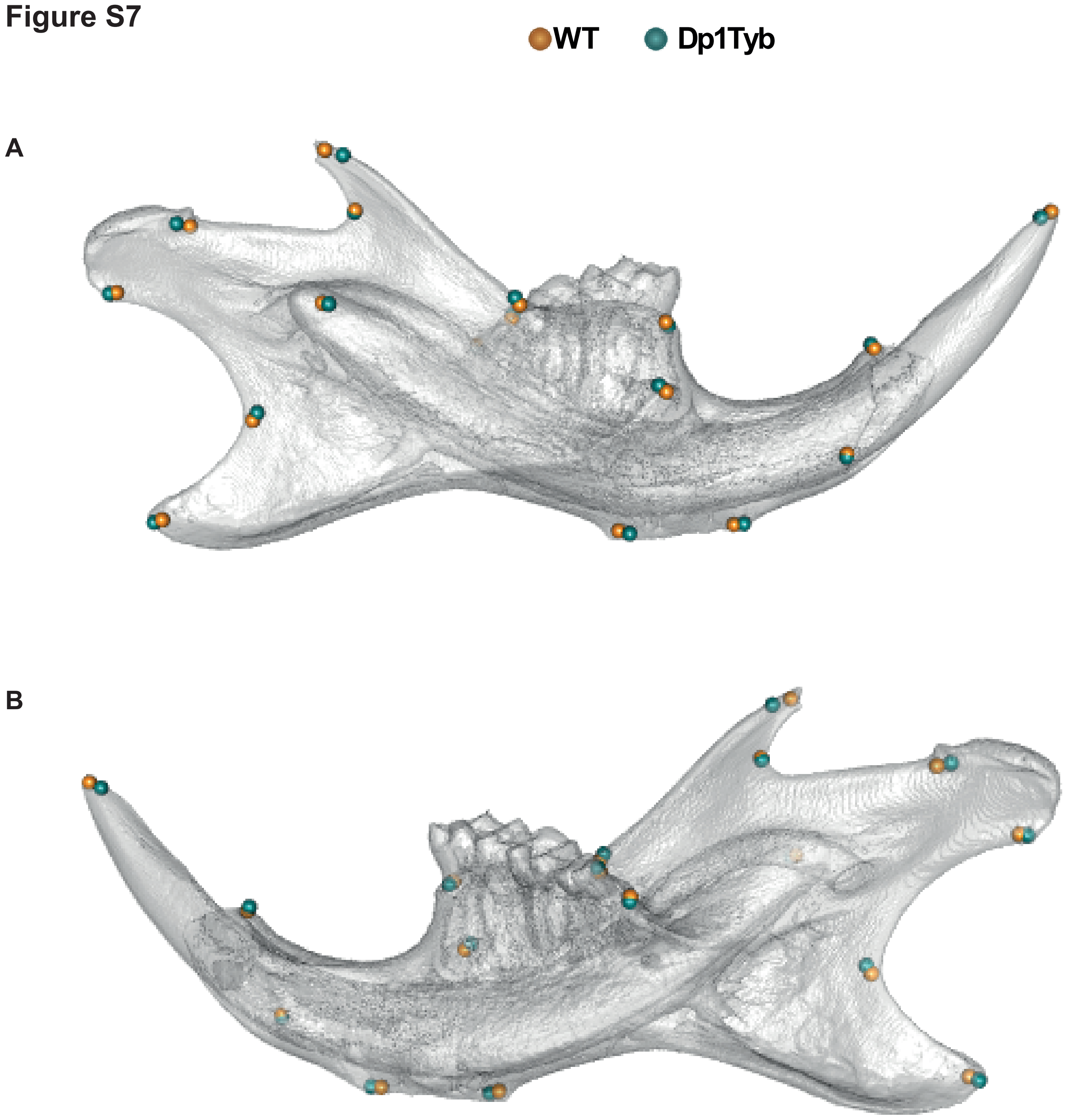

Fig. S7. Shape differences in Dp1Tyb mouse mandibles.
Mean landmark configurations of WT versus Dp1Tyb mandibles with global size differences removed, showing buccal (A) and lingual (B) views. Data are from a previous publication (Toussaint et al., 2021) where they were published under a CC-BY 4.0 license.

Figure S8

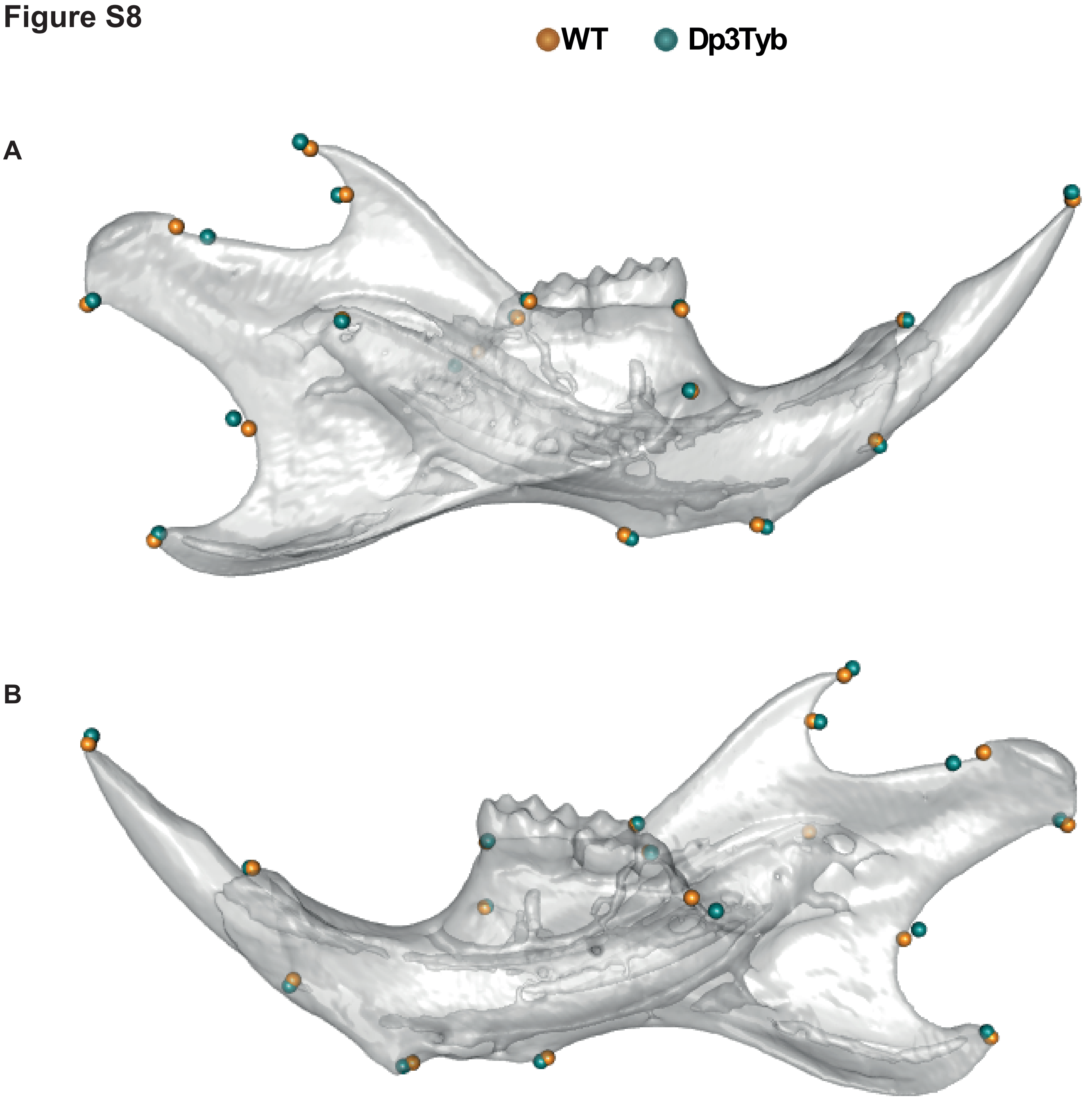

Fig. S8. Shape differences in Dp3Tyb mouse mandibles.
Mean landmark configurations of WT versus Dp3Tyb mandibles with global size differences removed, showing buccal (A) and lingual (B) views.

Development • Supplementary information

Figure S9

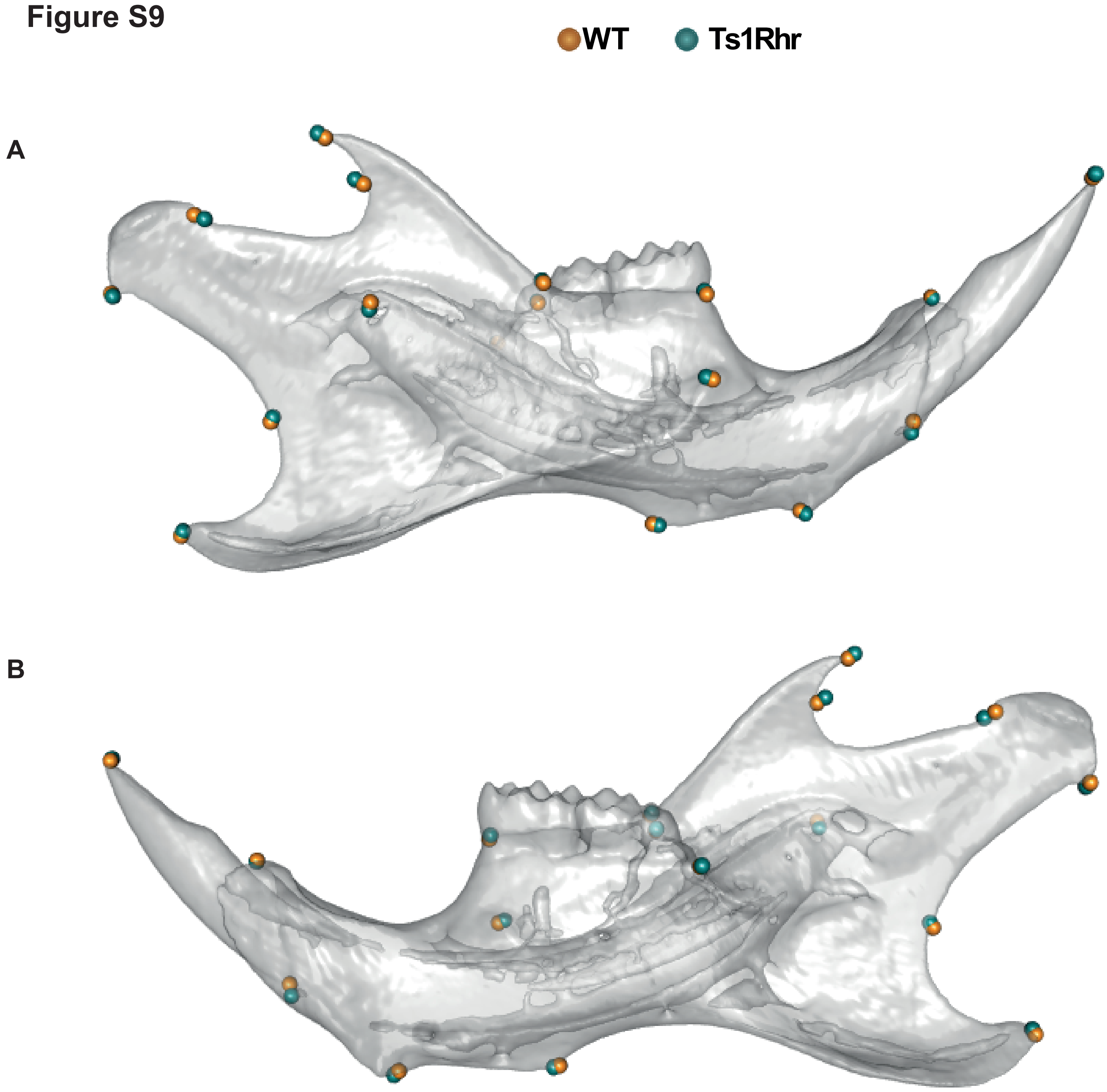

Fig. S9. Shape differences in Ts1Rhr mouse mandibles.
Mean landmark configurations of WT versus Ts1Rhr mandibles with global size differences removed, showing buccal (A) and lingual (B) views.

Figure S10

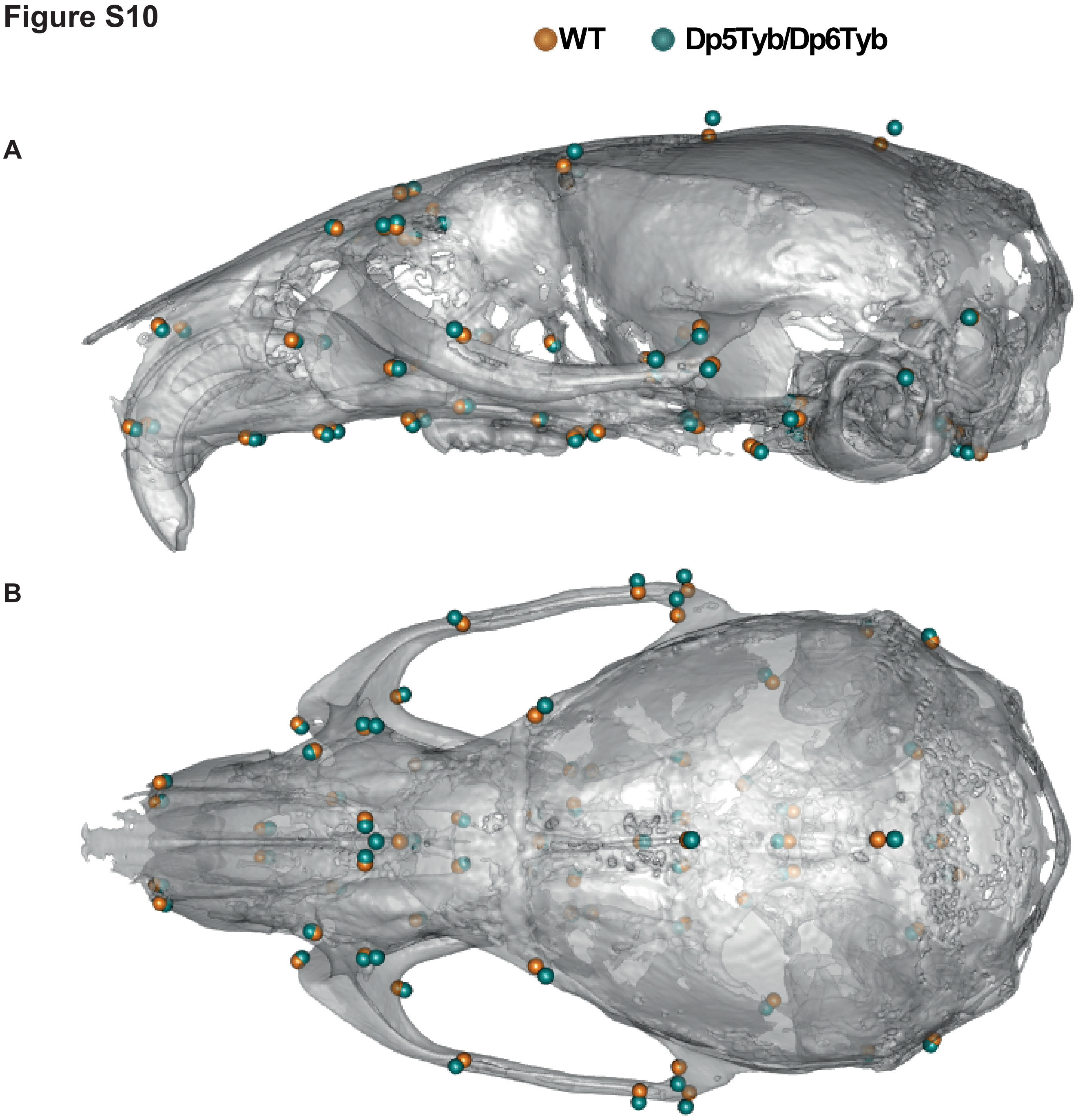

Fig. S10. Shape differences in Dp5Tyb/Dp6Tyb mouse crania.
Mean landmark configurations of WT versus Dp5Tyb/Dp6Tyb crania with global size differences removed, showing lateral (A) and superior (B) views.

Figure S11

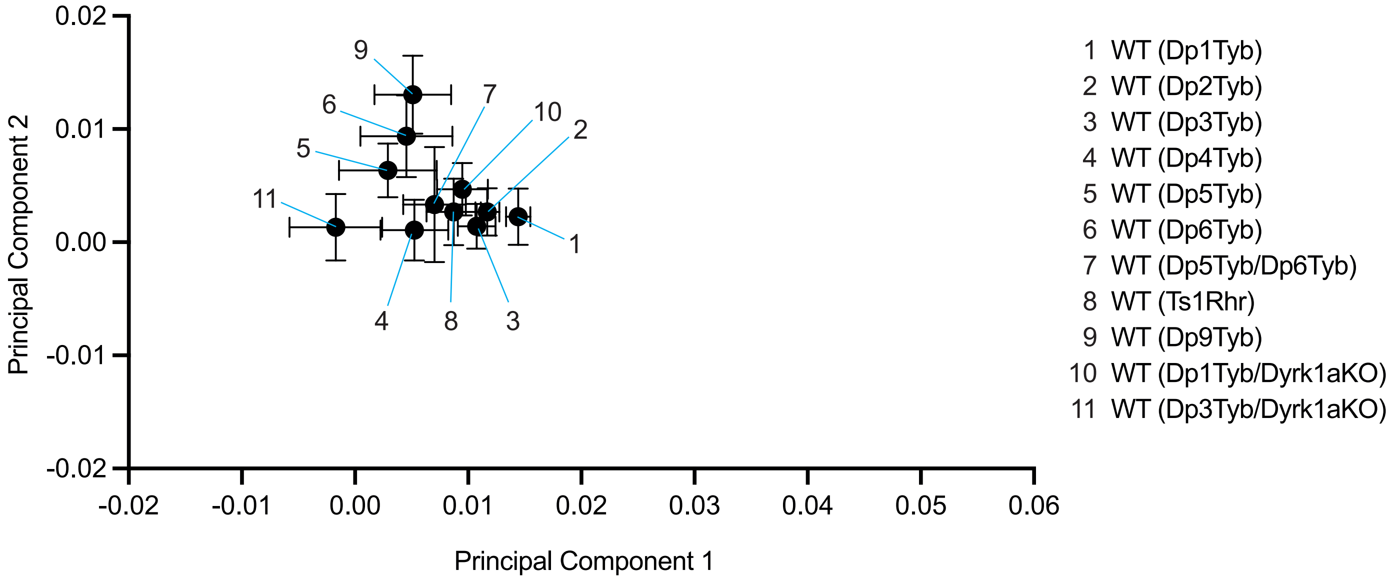

Fig. S11. PCA of WT cohorts.

PCA plot of cranial shapes of the WT control mice for each of the indicated mutant mouse strains, showing mean PCA values (± s.e.m).

Development • Supplementary information

Figure S12

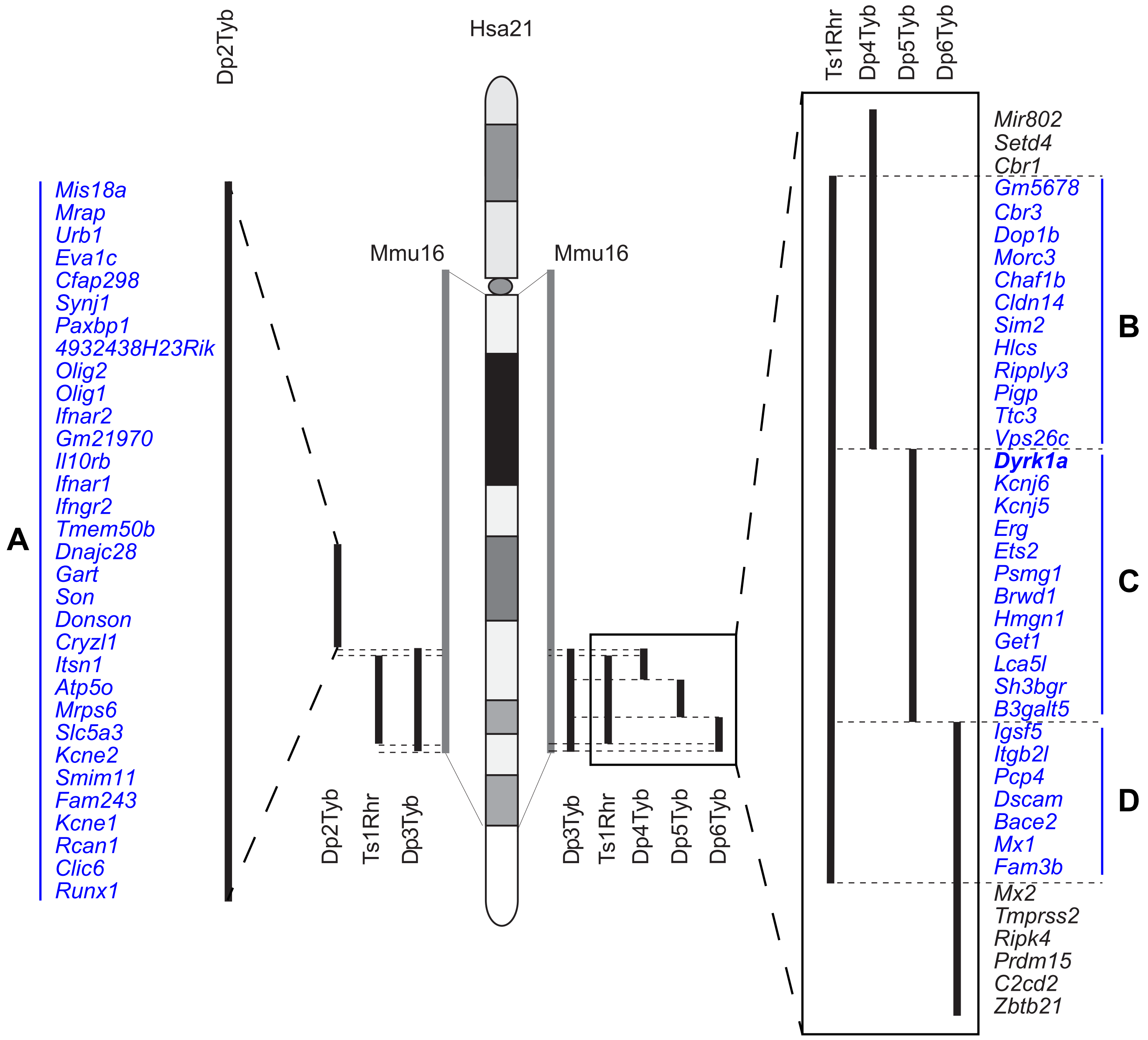

Fig. S12. Regions of Hsa21 containing dosage-sensitive genes causing craniofacial dysmorphology.
Diagram of Hsa21 showing main cytogenic regions (rectangles of different shades) and the centromere (oval). Grey bars indicate the Hsa21-orthologous region of Mmu16. Black lines on the right hand of the diagram indicate regions of Mmu16 duplicated in the Dp3Tyb, Ts1Rhr, Dp4Tyb, Dp5Tyb and Dp6Tyb mouse strains. Black lines on the left hand of the diagram indicate regions of Mmu16 duplicated in the Dp3Tyb, Ts1Rhr and Dp2Tyb strains. These regions are expanded showing all known protein coding genes within them and one microRNA gene (Mir802). Genes in blue lie within the four regions shown in this study to cause craniofacial phenotypes when present in three copies: regions A (Mis18a - Runx1), B (Gm5678 - Vps26c), C (Dyrk1a - B3galt5) and D (Igsf5 - Fam3b). The Dyrk1a gene (bold) is required in three copies for the full phenotype. Genes not within regions that cause craniofacial dysmorphology are in black.

Development • Supplementary information

Table S1. Raw Landmarking coordinates for crania.

This file contains the raw landmarking coordinates for the crania. The first line consists of headers identifying the coordinates in the order Landmark 1 x-coordinate, Landmark 1 y-coordinate, Landmark 1 z-coordinate, Landmark 2 x-coordinate, etc. Landmark numbers are as previously described (Hallgrímsson et al., 2007). Each subsequent line is preceded with a specimen identifier with the following format:

1st character: sex

2nd and 3rd characters: genotype, where

n = DpnTyb;
d = Dp5xDp6 double
mutant; r = Ts1Rhr;
k = Dp1TybDyrk1aKO;
e = Dp3TybDyrk1aKO

followed by (3rd character):

carrier (c) of the above genotype
wild type (w) sibling for the corresponding carrier

Remaining characters are the individual mouse numbers

Click here to download Table S1

Table S2. Raw Landmarking coordinates for mandibles.

Raw landmarking coordinates for the mandibles in the same format as Table S1.

Click here to download Table S2

Table S3. Centroid sizes of crania and mandibles from all mouse strains analysed in the genetic mapping panel.

Comparison of centroid sizes of crania and mandibles in 16-week old mice from all mutant mouse strains and their respective WT controls. *p*-values calculated using a t-test for all strains, except Dp1Tyb/Dyrk1aKO and Dp3Tyb/Dyrk1aKO for which a one-way ANOVA was used. NS, not significant (p-value > 0.05).

Strain	Structure	Mean±SEM centroid size (mm)		Centroid size difference between WT and mutant		*p*-value
		WT	Mutant	(mm)	%	
Dp1Tyb	Cranium	52.65±0.22	48.97±0.35	3.68	6.99	<0.0001
	Mandible	18.68±0.08	17.43±0.14	1.25	6.69	<0.0001
Dp9Tyb	Cranium	51.70±0.30	51.43±0.29	0.27	0.52	NS
	Mandible	18.29±0.11	18.19±0.05	0.10	0.55	NS
Dp2Tyb	Cranium	52.35±0.27	51.31±0.32	1.04	1.99	0.0254
	Mandible	18.46±0.06	18.04±0.12	0.42	2.28	0.0061
Dp3Tyb	Cranium	52.32±0.29	51.22±0.37	1.10	2.10	0.0336
	Mandible	18.50±0.07	18.08±0.11	0.42	2.27	0.0041
Dp4Tyb	Cranium	51.72±0.28	51.51±0.26	0.21	0.41	NS
	Mandible	18.29±0.07	18.04±0.09	0.25	1.37	0.0383
Dp5Tyb	Cranium	51.82±0.28	52.11±0.26	0.29	0.56	NS
	Mandible	18.45±0.08	18.29±0.08	0.16	0.87	NS
Dp6Tyb	Cranium	51.38±0.47	51.81±0.27	0.43	0.84	NS
	Mandible	18.22±0.13	18.25±0.10	0.03	0.16	NS
Ts1Rhr	Cranium	52.17±0.24	50.65±0.43	1.52	2.91	0.0068
	Mandible	18.48±0.07	18.02±0.10	0.46	2.49	0.0013
Dp5Tyb/Dp6Tyb	Cranium	52.17±0.23	51.82±0.32	0.35	0.67	NS
	Mandible	18.51±0.10	18.17±0.08	0.34	1.84	0.0113
Dp1Tyb/*Dyrk1a*KO	Cranium	52.03±0.26	50.38±0.44	1.65	3.17	0.0061
	Mandible	18.36±0.08	17.66±0.10	0.70	3.81	<0.0001
Dp3Tyb/*Dyrk1a*KO	Cranium	52.81±0.23	52.28±0.27	0.53	1.00	NS
	Mandible	18.73±0.05	18.53±0.06	0.20	1.07	NS

Table S4. Percentage variance accounted for by principal components.

Principal Component Analysis (PCA) carried out on landmark coordinates of crania and mandibles from the 11 different sets of 16-week old mice using MorphoJ. 68 and 17 landmarks were used for crania and mandibles, respectively. The tables show the percentage variance accounted for by each principal component in the PCA of each of 11 sets of data, with separate sheets for crania and mandibles respectively.

Click here to download Table S4

Development • Supplementary information

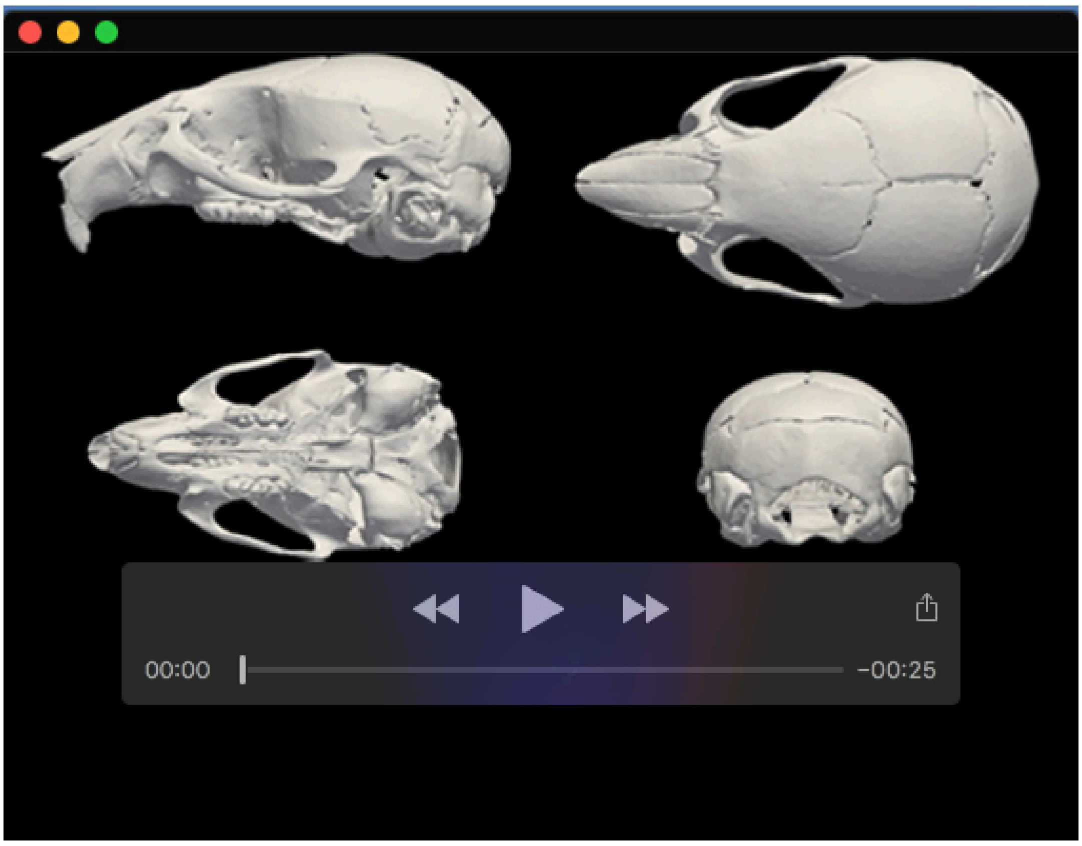

Movie 1. Cranial morph between WT and Dp1Tyb skulls at P21 with size regressed out.

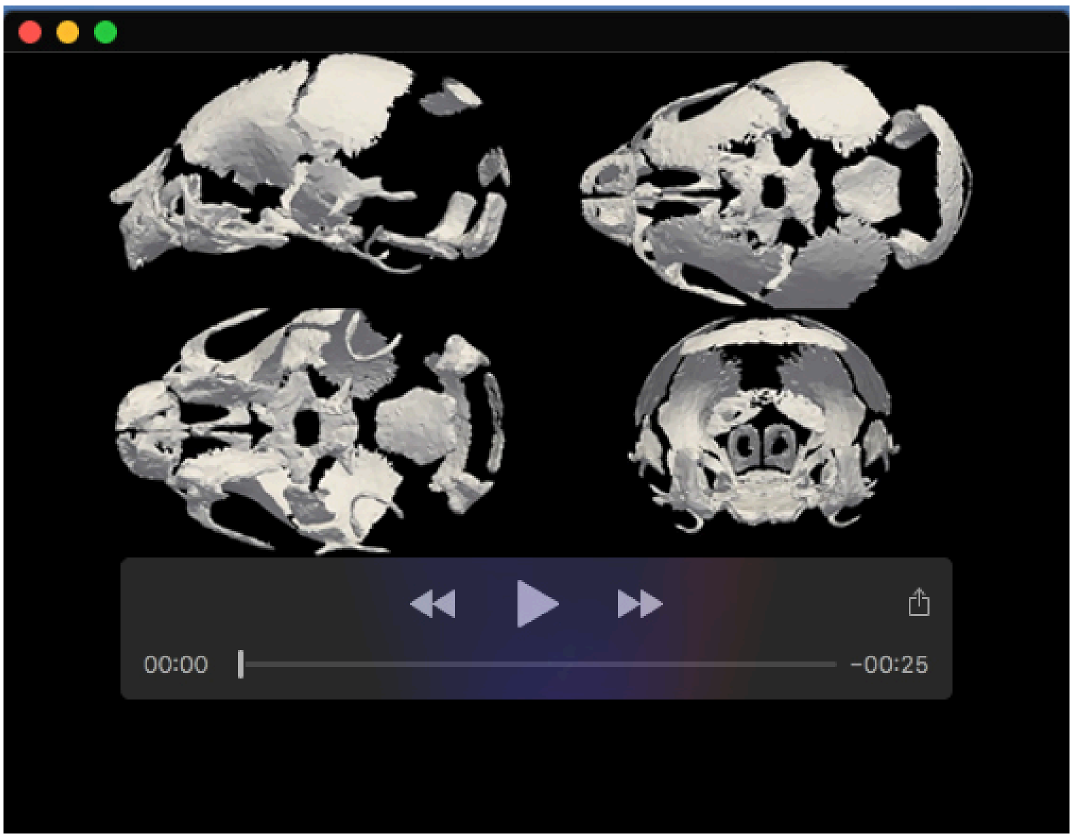

Movie 2. Cranial morph between WT and Dp1Tyb skulls at E18.5 with size regressed out.

Development • Supplementary information
