# Supplementary material for: Craniofacial dysmorphology in Down syndrome is caused by increased dosage of Dyrk1a and at least three other genes
Source: Development. 2023 Apr 26;150(8):dev201077. doi: 10.1242/dev.201077 (PMC10163349; doi:10.1242/dev.201077)
Supplement: Supplementary file 1 [file develop-150-201077_review_history.pdf]

Craniofacial dysmorphology in Down Syndrome is caused by increased dosage of Dyrk1a and at least three other genes

Yushi Redhead, Dorota Gibbins, Eva Lana-Elola, Sheona Watson-Scales, Lisa Dobson, Matthias Krause, Karen Liu, Elizabeth Fisher, Jeremy Green and Victor Tybulewicz
DOI: 10.1242/dev.201077

Editor: Liz Robertson

Review timeline
Original submission: 27 June 2022
Editorial decision: 19 July 2022
First revision received: 10 February 2023
Accepted: 21 March 2023

Original submission

First decision letter

MS ID#: DEVELOP/2022/201077

MS TITLE: Craniofacial dysmorphology in Down Syndrome is caused by increased dosage of Dyrk1a and at least three other genes

AUTHORS: Yushi Redhead, Dorota Gibbins, Eva Lana-Elola, Sheona Watson-Scales, Lisa Dobson, Matthias Krause, Karen Liu, Elizabeth Fisher, Jeremy Green, and Victor Tybulewicz

I have now received all the referees' reports on the above manuscript, and have reached a decision. The referees' comments are appended below, or you can access them online: please go to BenchPress and click on the 'Manuscripts with Decisions' queue in the Author Area.

As you will see, the referees express considerable interest in your work, but have some significant criticisms and recommend a substantial revision of your manuscript before we can consider publication. If you are able to revise the manuscript along the lines suggested, which may involve further experiments, I will be happy receive a revised version of the manuscript. Your revised paper will be re-reviewed by one or more of the original referees, and acceptance of your manuscript will depend on your addressing satisfactorily the reviewers' major concerns. Please also note that Development will normally permit only one round of major revision. If it would be helpful, you are welcome to contact us to discuss your revision in greater detail. Please send us a point-by-point response indicating your plans for addressing the referee’s comments, and we will look over this and provide further guidance.

Please attend to all of the reviewers' comments and ensure that you clearly highlight all changes made in the revised manuscript. Please avoid using 'Tracked changes' in Word files as these are lost in PDF conversion. I should be grateful if you would also provide a point-by-point response detailing how you have dealt with the points raised by the reviewers in the 'Response to Reviewers' box. If you do not agree with any of their criticisms or suggestions please explain clearly why this is so.

Reviewer 1

Advance summary and potential significance to field

© 2023. Published by The Company of Biologists under the terms of the Creative Commons Attribution License (https://creativecommons.org/licenses/by/4.0/).

The current manuscript by Redhead et al reports about the genetic dissection of craniofacial dysmorphism in Down syndrome partial model. The authors used DS models for the mouse chromosome 16 region covering a part of the region homologous to human chromosome 21 in the mouse and performed a morphological analysis of the adult cranium and mandible. They found that at least four loci, including Dyrk1a are involved and that bones from neural cell crest are the most affected for craniofacial changes. Then the authors analysed further the developmental origins of some changes observed in the Dp1Tyb and make interesting genetic link between Dyrk1a dosage, neural cell crest proliferation. The study is of interest and bring new genetic knowledge to the field of DS research.

Comments for the author

The current manuscript by Redhead et al reports about the genetic dissection of craniofacial dysmorphism in Down syndrome partial model. The authors used DS models for the mouse chromosome 16 region covering a part of the region homologous to human chromosome 21 in the mouse and performed a morphological analysis of the adult cranium and mandible. They found that at least four loci including Dyrk1a, are involved and that bones from neural cell crest are the most affected for craniofacial changes. Then the authors analysed further the developmental origins of some changes observed in the Dp1Tyb and make interesting genetic link between Dyrk1a dosage, neural cell crest proliferation.
The study is of interest and bring new genetic knowledge to the field of DS research.
Nevertheless, more detailed description of the craniofacial differences should be given. Currently the description of phenotypes about form and shape of the skull and mandibles can only be appreciated in a few figure (like figure 3 about shape). In addition, I would encourage revision to solve the concerns and comments below

1) first the low number of samples used (5 females and 5 males) even though the lines are on the same genetic background this number of 10 mixing both sexes is low considering some sexual dysmorphism already described some of the authors in Toussaint et al 2021 (doi:10.1242/dev.188631). Did the author correct for any sex difference observed between males and females? Can they indicate the position of samples from both sexes in the figures 1 and 2?
2) In the figures 1 and 2, the authors present Principal component analysis of the cranium/mandible with two different forms in A and B, and comment in the text the altered size and shape. They must show where the most important changes are? May be in a better figure 3 by showing landmark (at moment almost invisible in fig 3) and distances that are contributing to the changes.
3) Table S1 is not really informative here. More importantly would be to get access to the position of the landmark with a table of landmark for each individual used in the study so that the analysis can be redone. A need for open science…
4) In addition, they should indicate on the PCA the contribution of the dimension to explain the variances observed… and additional representation of correlation circle showing the contribution of specific “distances” to the PCA dimension may be very helpful especially while comparing all the models in figure 4.
5) It would be recommended to report here the same graphs with the Dp1Yey to better appreciate the difference rather than to try to estimate the difference with the results shown in Toussaint et al 2021.
6) I would also recommend to do a global comparison of the “wt” from the different lines that corresponds to the same genetic background to appreciate the cohort effects in a supplementary file.
7) Regarding the effect observed in the cranium of Ts1Rhr, qualified here to be with “altered shape and decrease size” (p5) similar to that seen in Dp3Tyb, seems to be in contradiction with the previous study done by Olson et al 2004 showing larger distances between many landmarks in the cranium and the mandibles. These different observations should be discussed with a more detailed figure really highlighting the distance affected in both models.
8) Fig3A is not easy to read in its current forms. Landmarks are small and the quality of the figures is low.
9) It would be may nice to consider to use a discriminant analysis to calculate the best discriminating components for groups that are genetically defined. The authors have all the data to do that and strongly support their hypothesis with more clear description of the phenotypes (not only small and large) and better definition of the role at least the 4 loci, suspected to be involved on Mmu16.

© 2023. Published by The Company of Biologists under the terms of the Creative Commons Attribution License (https://creativecommons.org/licenses/by/4.0/).

10) The results of the analysis of DYRK1A dosage effect in Dp1Tyb and Dp3Tyb is of interest but the results presented mainly in figure 5 are just about lack of multivariate analysis done in Principal component analysis with the same limitation described before (no explanation about the variance explained in dimension and factor involved). Discriminant analysis would have at least point to more detailed results. In addition, the contribution of DYRK1A overdosage alone as described for the brain in Guedj et al 2009.
11) I would be interesting to include the two combination of models Dp1Tyb Dp3Tyb with Dyrk1a mutation in a general figure comparable to figure 4 to compare the contribution with the other genetic configuration alone and to appreciate any cohort effects.
12) Regarding the results about the role of DYRK1A dosage in the decreased proliferation of neural crest cells (NC), the authors should consider to show increase of Dyrk1a expression in the NC to confirm the genetic analysis and their hypothesis. In addition, they should also discuss discrepancies with previous study on migrating NC in DS models
13) According to the reference (Fotaki et al., 2002) the Dyrk1a<tm1Mla> mice (Dyrk1a+/-, Dyrk1aKO) were generated on a C57BL/6–129Ola genetic background. Can the author confirm the genetic background of Dyrk1a Ko mice used when combining with Dp1Tyb and Dp3Tyb duplications? In addition, can the author comment on the transmission rate of the different genotype from the Dp1Tyb-Dyrk1aKo in cis?
14) It would be nice to see a clear figure with specific change by models… an improved figure 3…

<u>Reviewer 2</u>

Advance summary and potential significance to field

This manuscript describes work to identify the gene or genes responsible for craniofacial defects in a mouse model of Downs' Syndrome (DS). Among other defects, humans with trisomy 21 have a flatter skull, a wider face and a smaller jaw. Several mouse models of DS exist, in which an additional copy of the orthologues of many or most of the genes on HSA21 are present. The craniofacial dysmorphologies seen in DS patients appear to be well modelled in these mice strains.

The Dp1Tyb model, previously described, is an internal duplication on MMU16 which is trisomic for 142 of the ~230 genes on HSA21. Clever genetic manipulation has generated 7 derivatives of the Dp1Tyb mice in which the duplicated segment of MMU16 is reduced in different lines, so reducing number of genes with increased dosage. These have been previously pubished in Disease Models and Mechanisms in which the effect of the trisomies on cardiac development was described.

In this work the authors performed microCT on each strain, and carried out morphometric analyses. 3 strains, each with non-overlapping segments, had craniofacial abnormalities, revealed by PCA of multiple measurements on individual mice. Additional mapping, including combining duplicated segments suggests at least 4 genes, one mapping to each segment, were responsible for abnormalities in the mouse model.

One critical region contains only 12 genes. One of these, Dyrk1a, has been shown in multiple previous studies to result in DS-like phenotypes, including craniofacial defects, when an additional copy is present. In this manuscript the authors carried out an elegant genetic cross whereby a knock-out of Dyrk1a was placed opposite a partial MMU16 duplication, thus generating mice with three copies of all genes in the duplication, except for Dyrk1a. Analysis of these mice finds the KO partially rescues the craniofacial phenotype of Dp1Tyb and fully rescues the Dp3Tyb mouse phenotype, which contains 37 genes in additikon to Dyrk1a.

Considerable morphometric analyses of embryonic and young mice with the large Dp1Tyb duplication shows the dysmorphology is present at E16.5. They find much of the defect can be attributed to a reduction in the bones derived from the neural crest. Focussing on one of these, the frontal bone, they find a cellular deficit at E13.5. Critically this defect was absent when Dp1Tyb was placed in combination with a Dyrk1a KO, suggesting this particular defect was due to an additional copy of Dyrk1a (Again using elegant genetics as the mice carrying Dp1Tyb are poor breeders). However, further analysis quantifying gene expression in situ finds that Msx2 and Osterix, critical genes for bone development, were not reduced in expression, but Runx2 was

© 2023. Published by The Company of Biologists under the terms of the Creative Commons Attribution License (https://creativecommons.org/licenses/by/4.0/).

reduced. However this reduction was still seen in the Dp1Tyb/Dyrk1a KO embryos, despite the frontal bone phenotype being rescued, raising further questions.

Defects in neural crest derived tissue could be due to reduced cell number or to reduced migration of the cells away dfeom the neural tube. The authors cultured neural tubes and analysed cells migrating from the cultures. They find no difference in speed or directionality. They did not assay the number/proliferation of these cells. They did, however, use Sox10 expression to identify neural crest cells in E9.5 mutant and control embryos and DAPI to identify mitotic figures and thus dividing cells in that population. They find a reduction in proliferation in the Dp1Tyb embryos. This could be due to any of many genes in this duplication, but by introducing the Dyrk1a KO they find restoring copy number of Dyrk1a to diploid restores neural crest proliferatino to normal.

They conclude by discussing plausible models through which Dyrk1a may act on neural crest cells.

Comments for the author

Overall, the work is well done. Some of their findings have been previously reported by others, in particular the putative role of an additional copy of Dyrk1a in DS cranifacial dysmorphology. This manuscript indicates that Dyrk1a is not the only gene contributing to the phenotype and delves deeper into the develpmental basis of the defect.

Some minor points:

1. Can the authors draw deeper parallels between the skull and mandibular developmental abnormalities they find in the mouse model and the presumed defects in the equivalent DS patient dysmorphologies?

2. In examining neural crest in their models, why measure only proliferation via mitotic cells? One would expect to see an obervable difference in Sox10+ cell numbers. Has this been measured?

3. Genetic background is likely to influence subtle phenotype differences. They carefully describe the genetic backgrounds of the duplication mice as being congenic on C57BL/6J. However I don't see a description of the genetic background of the Dyrk1a KO mice, except the statement that all mice were backcrossed to C57BL/6J for 10 generations. Does this apply to the KO? The KO mice they use, from Fotaki et al, were derived on a 129P2 (E14 ES cell) background and even with extensive backcrossing there will remain a substantial amount of 129P2 genome around the Dyrk1a locus (linked segments reduce very slowly in backcrossing unlike unlinked genome). It is thus possible that there might be differences in gene expression or function of genes other than Dyrk1a in the linked 129P2 genome that ameliorate the craniofacial phenotype in the rescue cross. Although unlikely it should be considered.

First revision

Author response to reviewers' comments

Response to Reviewers

Reviewer 1. Advance Summary and Potential Significance to Field:
The current manuscript by Redhead et al reports about the genetic dissection of craniofacial dysmorphism in Down syndrome partial model. The authors used DS models for the mouse chromosome 16 region covering a part of the region homologous to human chromosome 21 in the mouse and performed a morphological analysis of the adult cranium and mandible. They found that at least four loci, including Dyrk1a, are involved and that bones from neural cell crest are the most affected for craniofacial changes. Then the authors analysed further the developmental origins of some changes observed in the Dp1Tyb and make interesting genetic link between Dyrk1a dosage, neural cell crest proliferation. The study is of interest and bring new genetic knowledge to the field of DS research.

© 2023. Published by The Company of Biologists under the terms of the Creative Commons Attribution License (https://creativecommons.org/licenses/by/4.0/).

<u>**Reviewer 1. Comments for the Author:**</u>

The current manuscript by Redhead et al reports about the genetic dissection of craniofacial dysmorphism in Down syndrome partial model. The authors used DS models for the mouse chromosome 16 region covering a part of the region homologous to human chromosome 21 in the mouse and performed a morphological analysis of the adult cranium and mandible. They found that at least four loci, including Dyrk1a, are involved and that bones from neural cell crest are the most affected for craniofacial changes. Then the authors analysed further the developmental origins of some changes observed in the Dp1Tyb and make interesting genetic link between Dyrk1a dosage, neural cell crest proliferation. The study is of interest and bring new genetic knowledge to the field of DS research.

Nevertheless, more detailed description of the craniofacial differences should be given. Currently the description of phenotypes about form and shape of the skull and mandibles can only be appreciated in a few figure (like figure 3 about shape). In addition, I would encourage revision to solve the concerns and comments below.

1) first the low number of samples used (5 females and 5 males) even though the lines are on the same genetic background this number of 10 mixing both sexes is low considering some sexual dysmorphism already described some of the authors in Toussaint et al 2021 (doi:10.1242/dev.188631). Did the author correct for any sex difference observed between males and females? Can they indicate the position of samples from both sexes in the figures 1 and 2?

This is an important point, however in this instance no correction for sex differences was needed because the differences between female and male were small. Our previous study of Dp1Tyb skulls showed no sex difference in the shape of the cranium and only a subtle difference in the shape of the mandible (Figure 2, Toussaint et al, 2021). Importantly, for both cranium and mandible the effect of genotype was stronger than sex both for Dp1Tyb mice and the other strains. This point is now noted in the Results on page 5, paragraph 1.

As requested, we have edited the PCA plots in Figures 1, 2, 5 and 7 to distinguish female and male subjects. For the study of E16.5 and E18.5 embryos (Figure 8) we did not record the sex of the embryos.

2) In the figures 1 and 2, the authors present Principal component analysis of the cranium/mandible with two different forms in A and B, and comment in the text the altered size and shape. They must show where the most important changes are? May be in a better figure 3 by showing landmark (at moment almost invisible in fig 3) and distances that are contributing to the changes.

To address this point, we have generated 10 new Supplementary Figures (Figures S1-S10) with high-resolution versions of the images of the crania and mandibles shown in Figure 3A-M. These versions make the shape differences between mutants and controls much clearer. The reviewer asks about distances on these plots. Adding scale bars is not meaningful in this case, because to illustrate the shape changes, these images have had size differences regressed out. In paragraph 2 on page 6 we have added some extra detail to the description of the phenotype and point the reader to our earlier paper (Toussaint et al, 2021) in which the phenotype is described in greater detail.

3) Table S1 is not really informative here. More importantly would be to get access to the position of the landmark with a table of landmark for each individual used in the study so that the analysis can be redone. A need for open science…

As requested, we have generated two new Tables (Table S1 and S2) with the raw landmarking coordinates. Readers could use these to redo any analysis or perform any additional analysis they choose.

4) In addition, they should indicate on the PCA the contribution of the dimension to explain the variances observed… and additional representation of correlation circle showing the contribution of specific "distances" to the PCA dimension may be very helpful especially while comparing all the models in figure 4.

© 2023. Published by The Company of Biologists under the terms of the Creative Commons Attribution License (https://creativecommons.org/licenses/by/4.0/).

As requested, we have generated a new Table S4 showing the percentage variance accounted for by each principal component dimension for each principal component analysis of crania and mandibles. Regarding presenting the specific contributions of individual landmarks to the principal components in these analyses, we believe this is not particularly relevant to the genetic mapping or the cellular basis of the phenotypes that are the focus of this study. Our previous publication (Toussaint et al, 2021) provides detailed heatmaps and morphs that show where the Dp1Tyb-associated changes from wild-type are localised, while in this study the morphological changes are shown in the images in Figure 3 and their high-resolution counterparts in Figures S1-S10. Nonetheless, with the raw landmark data that we now provide in Tables S1 and S2, it will be open to others to re-use these data for further analysis.

5) It would be recommended to report here the same graphs with the Dp1Yey to better appreciate the difference rather than to try to estimate the difference with the results shown in Toussaint et al 2021.

We assume the reviewer is referring to our analysis of the morphological changes in Dp1Tyb mice in Toussaint et al (Dp1Tyb and Dp1Yey mice have an extra copy of the same region of Mmu16). As requested, we have added PCA plots and centroid sizes of the changes in Dp1Tyb crania and mandibles to Figures 1 and 2, allowing direct comparisons to the other mutant strains.

6)I would also recommend to do a global comparison of the “wt” from the different lines that corresponds to the same genetic background to appreciate the cohort effects in a supplementary file.

As requested, we have generated a new Figure S11 with a PCA plot comparing the WT cohorts from the different lines. This was made as part of generating the new version of Figure 4A which shows all the strains on a single PCA plot. Thus, the graph with the WT cohorts is on the same PCA scale as Figure 4A.

7) Regarding the effect observed in the cranium of Ts1Rhr, qualified here to be with “altered shape and decrease size” (p5) similar to that seen in Dp3Tyb, seems to be in contradiction with the previous study done by Olson et al 2004 showing larger distances between many landmarks in the cranium and the mandibles. These different observations should be discussed with a more detailed figure really highlighting the distance affected in both models.

As we report, the shape changes in Dp3Tyb and Ts1Rhr mice are very similar in our hands - both strains show cranial doming and a shortening front to back. This can be seen in the images in Figures 3E and 3F and is clearer in the new high-resolution versions in Figures S3 and S4. Furthermore, the all-strain PCA plot in Figure 4A shows that the shape changes in Dp3Tyb and Ts1Rhr crania are very similar. The size changes in Dp3Tyb and Ts1Rhr crania are also similar - a reduction of 2.1% - 2.9% (Table S3).

The reviewer refers to a study by Olson et al 2004 in which they report that Ts1Rhr crania have increased inter-landmark distances between the front and back of the crania, so, yes, we agree that there is a contradiction as the reviewer points out. Our data indeed show the opposite of what Olson et al. reported - we find that Ts1Rhr skulls are shorter back to front, showing brachycephaly. The reason for the difference between our study and that of Olson et al is unclear. One possibility is that while we conducted all our analyses an inbred C57BL/6J background, Olson et al were working on a B6xC3H segregating F2 background. We have added new text on page 6 to flag up and discuss this discrepancy.

8) Fig3A is not easy to read in its current forms. Landmarks are small and the quality of the figures is low.

To address this point, we have generated 10 new Supplementary Figures (Figures S1-S10) consisting of high-resolution versions of the images of the crania and mandibles shown in Figure 3A-M. These versions make the shape differences between mutants and controls much clearer.

9) It would be may nice to consider to use a discriminant analysis to calculate the best discriminating components for groups that are genetically defined. The authors have all the

© 2023. Published by The Company of Biologists under the terms of the Creative Commons Attribution License (https://creativecommons.org/licenses/by/4.0/).

data to do that and strongly support their hypothesis with more clear description of the phenotypes (not only small and large) and better definition of the role at least the 4 loci, suspected to be involved on Mmu16.

While a discriminant analysis might highlight a hierarchy of differences between changes in different landmarks, this would not inform either the genetic mapping or the underlying cellular mechanisms which are the focus of our work. Therefore, we feel that this is beyond the scope of the present paper. Nonetheless, we have added more detail to our description of the shape changes seen in Dp1Tyb mice and the strains with shorter trisomic regions, which, with the exception of Dp6Tyb, show the same changes as Dp1Tyb albeit smaller in magnitude.

10) The results of the analysis of DYRK1A dosage effect in Dp1Tyb and Dp3Tyb is of interest but the results presented mainly in figure 5 are just about lack of multivariate analysis done in Principal component analysis with the same limitation described before (no explanation about the variance explained in dimension and factor involved). Discriminant analysis would have at least point to more detailed results. In addition, the contribution of DYRK1A overdosage alone as described for the brain in Guedj et al 2009.

We found that removing one copy of Dyrk1a partially rescues the Dp1Tyb phenotype and fully rescues the Dp3Tyb cranial shape phenotype. Moreover, as is clear in Fig.4, the Dp1Tyb/Dyrk1aKO crania lie on much the same shape trajectory as going from wild type to Dp1Tyb so are not going to uncover a novel subset of morphometric distance changes unless these were proportionally very small. At this point we feel that this level of granular analysis would be pushing the limit of what the sample number can provide adequate support for. Quite separately, we also believe that fine-grained discrimination of which landmarks or groups of landmarks are involved in these rescues would not not add to our understanding of either the genetics or the cellular mechanisms that are the focus of this paper. Nonetheless, any interested reader will now be able to perform further analysis using the raw landmark data provided in the supplementary files. As requested, we have also generated a new Table S4 showing the percentage variance accounted for by each principal component dimension for the PCA of the Dp1Tyb/Dyrk1aKO and Dp3Tyb/Dyrk1aKO strains.

11) I would be interesting to include the two combination of models Dp1Tyb, Dp3Tyb with Dyrk1a mutation in a general figure comparable to figure 4 to compare the contribution with the other genetic configuration alone and to appreciate any cohort effects.

As requested, we have generated a new version of Figure 4A in which we now include the Dp1Tyb/Dyrk1aKO and Dp3Tyb/Dyrk1aKO strains as part of the analysis. It shows that the Dyrk1aKO mutation rescues most of the shape phenotype of Dp1Tyb crania and fully rescues the Dp3Tyb shape phenotype.

12) Regarding the results about the role of DYRK1A dosage in the decreased proliferation of neural crest cells (NC), the authors should consider to show increase of Dyrk1a expression in the NC to confirm the genetic analysis and their hypothesis. In addition, they should also discuss discrepancies with previous study on migrating NC in DS models.

The reviewer raises an interesting question, which is whether the effects on neural crest derivatives are due to autonomous increase of duplicated gene expression or could arise through non-autonomous effects. We have carried out RNA-sequencing on multiple tissues from Dp1Tyb mice: mouse embryonic fibroblasts (Ahlfors et al 2019, Nat Comms), adult hippocampus (Lana-Elola et al, 2021, Dis Models Mechanism) and embryonic hearts (unpublished). In all cases we have seen increased expression of almost every duplicated gene, including Dyrk1a, with a mean increase of ~1.4, close to the expected 1.5-fold increase. Hence, we would strongly expect Dyrk1a expression to be elevated in all tissues in Dp1Tyb mice, including neural crest cells and their derivatives. However, a thorough analysis would constitute an entirely new project since it would need isolation of neural crest and derivatives at multiple stages and comparison with non-neural crest-derived tissues such as oral epithelium and intermingled mesenchymal mesoderm derivatives. We thank the reviewer for this idea and have alluded to this issue in new text in the Discussion in paragraph 2 on page 16.

© 2023. Published by The Company of Biologists under the terms of the Creative Commons Attribution License (https://creativecommons.org/licenses/by/4.0/).

The difference between our results on neural crest cell migration in Dp1Tyb embryos (no effect) and the reported reduced migration in Ts65Dn neural crest cells (Roper et al, 2009) maybe due to the genetics of the Ts65Dn model. Ts65Dn mice have an extra copy of 46 genes that are not orthologous to Hsa21. Thus, these may have caused the decreased migration of the neural crest cells. We discuss this point on page 16, paragraph 3.

13) According to the reference (Fotaki et al., 2002) the Dyrk1a mice (Dyrk1a+/-, Dyrk1aKO) were generated on a C57BL/6-129Ola genetic background. Can the author confirm the genetic background of Dyrk1a Ko mice used when combining with Dp1Tyb and Dp3Tyb duplications? In addition, can the author comment on the transmission rate of the different genotype from the Dp1Tyb-Dyrk1aKo in cis?

All mouse strains were backcrossed for at least 10 generations to the C57BL/6J background as described in the Methods. This is also true for the two strains carrying the Dyrk1aKO mutation.

The Dp1Tyb/Dyrk1aKO mouse strain has the two mutations (Dp1Tyb and Dyrk1aKO) in cis on the same chromosome. Thus, they segregate together, and in crosses of Dp1Tyb/Dyrk1aKO to B6 mice, we recover Dp1Tyb/Dyrk1aKO and WT mice at the expected Mendelian ratio of 1:1. This is described in the Methods. As would be expected, we occasionally (1 in ~30 pups) see the two mutations separate, indicating chromosomal crossover during meiosis.

14) It would be nice to see a clear figure with specific change by models… an improved figure 3…

To address this point, we have generated 10 new Supplementary Figures (Figures S1-S10) with high-resolution versions of the images of the crania and mandibles shown in Figure 3A-M. These versions make the shape differences between mutants and controls much clearer.

<u>Reviewer 2. Advance Summary and Potential Significance to Field:</u>
This manuscript describes work to identify the gene or genes responsible for craniofacial defects in a mouse model of Downs' Syndrome (DS). Among other defects, humans with trisomy 21 have a flatter skull, a wider face and a smaller jaw. Several mouse models of DS exist, in which an additional copy of the orthologues of many or most of the genes on HSA21 are present. The craniofacial dysmorphologies seen in DS patients appear to be well modelled in these mice strains.

The Dp1Tyb model, previously described, is an internal duplication on MMU16 which is trisomic for 142 of the ~230 genes on HSA21. Clever genetic manipulation has generated 7 derivatives of the Dp1Tyb mice in which the duplicated segment of MMU16 is reduced in different lines, so reducing number of genes with increased dosage. These have been previously published in Disease Models and Mechanisms in which the effect of the trisomies on cardiac development was described.

In this work the authors performed microCT on each strain, and carried out morphometric analyses. 3 strains, each with non-overlapping segments, had craniofacial abnormalities, revealed by PCA of multiple measurements on individual mice. Additional mapping, including combining duplicated segments suggests at least 4 genes, one mapping to each segment, were responsible for abnormalities in the mouse model.

One critical region contains only 12 genes. One of these, Dyrk1a, has been shown in multiple previous studies to result in DS-like phenotypes, including craniofacial defects, when an additional copy is present. In this manuscript the authors carried out an elegant genetic cross whereby a knock-out of Dyrk1a was placed opposite a partial MMU16 duplication, thus generating mice with three copies of all genes in the duplication, except for Dyrk1a. Analysis of these mice finds the KO partially rescues the craniofacial phenotype of Dp1Tyb and fully rescues the Dp3Tyb mouse phenotype, which contains 37 genes in addition to Dyrk1a.

Considerable morphometric analyses of embryonic and young mice with the large Dp1Tyb duplication shows the dysmorphology is present at E16.5. They find much of the defect can be

© 2023. Published by The Company of Biologists under the terms of the Creative Commons Attribution License (https://creativecommons.org/licenses/by/4.0/).

attributed to a reduction in the bones derived from the neural crest. Focussing on one of these, the frontal bone, they find a cellular deficit at E13.5. Critically this defect was absent when Dp1Tyb was placed in combination with a Dyrk1a KO, suggesting this particular defect was due to an additional copy of Dyrk1a (Again using elegant genetics as the mice carrying Dp1Tyb are poor breeders). However, further analysis quantifying gene expression in situ finds that Msx2 and Osterix, critical genes for bone development, were not reduced in expression, but Runx2 was reduced. However this reduction was still seen in the Dp1Tyb/Dyrk1a KO embryos, despite the frontal bone phenotype being rescued, raising further questions.

Defects in neural crest derived tissue could be due to reduced cell number or to reduced migration of the cells away dfeom the neural tube. The authors cultured neural tubes and analysed cells migrating from the cultures. They find no difference in speed or directionality. They did not assay the number/proliferation of these cells. They did, however, use Sox10 expression to identify neural crest cells in E9.5 mutant and control embryos and DAPI to identify mitotic figures and thus dividing cells in that population. They find a reduction in proliferation in the Dp1Tyb embryos. This could be due to any of many genes in this duplication, but by introducing the Dyrk1a KO they find restoring copy number of Dyrk1a to diploid restores neural crest proliferation to normal.

They conclude by discussing plausible models through which Dyrk1a may act on neural crest cells.

<u>Reviewer 2. Comments for the Author:</u>
Overall, the work is well done. Some of their findings have been previously reported by others, in particular the putative role of an additional copy of Dyrk1a in DS craniofacial dysmorphology. This manuscript indicates that Dyrk1a is not the only gene contributing to the phenotype and delves deeper into the developmental basis of the defect.

Some minor points:
1. Can the authors draw deeper parallels between the skull and mandibular developmental abnormalities they find in the mouse model and the presumed defects in the equivalent DS patient dysmorphologies?

The comparison between the craniofacial changes in Dp1Tyb mice and humans with DS was discussed in detail in our previous publication describing the Dp1Tyb phenotype (Toussaint et al, 2021, Development). In this earlier publication we showed that Dp1Tyb mice show midfacial hypoplasia, occipital reduction, shortening of the skull along the anteroposterior axis (brachycephaly), and a reduction in the dimensions of the lower jaw (micrognathia), all phenotypes that are typical of humans with DS. Thus, we concluded that Dp1Tyb mice model the overall craniofacial abnormalities in DS. In the current study we demonstrate that Dp1Tyb mice have aberrant synchondroses, with evidence of premature fusion and mineralisation, and we hypothesise that this may underlie the midfacial hypoplasia. Interestingly, premature fusion of synchondroses has also been reported in humans with DS at 7 months of age, suggesting that this may also cause midfacial hypoplasia in the human condition. We discuss this on page 15, paragraph 3.

2. In examining neural crest in their models, why measure only proliferation via mitotic cells? One would expect to see an observable difference in Sox10+ cell numbers. Has this been measured?

We have shown that at E13.5 there are many fewer cells in Dp1Tyb frontal bone primordia, structures which are almost entirely neural crest derived. However, we have not counted the numbers of Sox10+ cells at the earlier E9.5 stage, where we only measured proliferation. We felt this was a natural stopping point in the project, allowing us to hypothesise that a Dyrk1a-dependent decrease in proliferation of NC-derived cells may contribute to the decreased size of the frontal bone primordia.

To take this work further, would require substantially more work. The reduced number of cells in Dp1Tyb frontal bone primordia at E13.5 could be due to many different causes, including decreased proliferation at earlier stages of development (such as E9.5), increased cell death, changes in rates of differentiation, or combinations of any of these. Disentangling these various possibilities would require carefully analysing cell numbers, proliferation, and death at multiple time points through

© 2023. Published by The Company of Biologists under the terms of the Creative Commons Attribution License (https://creativecommons.org/licenses/by/4.0/).

development. This would be a substantial project in its own right, and we feel is beyond the scope of the current study.

3. Genetic background is likely to influence subtle phenotype differences. They carefully describe the genetic backgrounds of the duplication mice as being congenic on C57BL/6J. However I don't see a description of the genetic background of the Dyrk1a KO mice, except the statement that all mice were backcrossed to C57BL/6J for 10 generations. Does this apply to the KO? The KO mice they use, from Fotaki et al, were derived on a 129P2 (E14 ES cell) background and even with extensive backcrossing there will remain a substantial amount of 129P2 genome around the Dyrk1a locus (linked segments reduce very slowly in backcrossing unlike unlinked genome). It is thus possible that there might be differences in gene expression or function of genes other than Dyrk1a in the linked 129P2 genome that ameliorate the craniofacial phenotype in the rescue cross. Although unlikely it should be considered.

As with all the strains, the Dyrk1a KO mutation was also backcrossed (>10 generations) to C57BL/6J. The reviewer is correct that there will always be a bit of the genome around this mutation that will be derived from the donor strain (129P2 in this case) on which the mutation was established. This is also true for the panel of DpXTyb strains which were generated by targeting loxP sites into the genome of ES cells on a 129P2 background followed by Cre-mediated recombination to generate the duplication strains (Lana-Elola et al, 2016, eLife). Thus, these mice inevitably also carry a small portion of 129P2 genome around the duplication mutation. As the reviewer points out, it is possible that these small portions of the 129P2 genome may affect the phenotype. We have added a discussion of this point to the Methods (page 18).

<u>Second decision letter</u>

MS ID#: DEVELOP/2022/201077

MS TITLE: Craniofacial dysmorphology in Down Syndrome is caused by increased dosage of Dyrk1a and at least three other genes

AUTHORS: Yushi Redhead, Dorota Gibbins, Eva Lana-Elola, Sheona Watson-Scales, Lisa Dobson, Matthias Krause, Karen Liu, Elizabeth Fisher, Jeremy Green, and Victor Tybulewicz
ARTICLE TYPE: Research Article

I am happy to tell you that your manuscript has been accepted for publication in Development, pending our standard ethics checks.

<u>Reviewer 1</u>

Advance summary and potential significance to field

The paper reports about the analysis of genotype and phenotype relationship in partial down syndrome mouse models. It unravels a complex genetic with a key driver gene, whose overdosage is important for the phenotypes but with the contribution of other genes located in the mouse chromosome 16.

Comments for the author

The authors addressed all the questions raised within the revision of their manuscript.

<u>Reviewer 2</u>

Advance summary and potential significance to field

As before

© 2023. Published by The Company of Biologists under the terms of the Creative Commons Attribution License (https://creativecommons.org/licenses/by/4.0/).

Comments for the author

The revised manuscript has addressed the points which I raised, and, as far as I can tell addresses those of the other reviewer

© 2023. Published by The Company of Biologists under the terms of the Creative Commons Attribution License (https://creativecommons.org/licenses/by/4.0/).
